# Iron Oxide@Mesoporous Silica Core-Shell Nanoparticles as Multimodal Platforms for Magnetic Resonance Imaging, Magnetic Hyperthermia, Near-Infrared Light Photothermia, and Drug Delivery

**DOI:** 10.3390/nano13081342

**Published:** 2023-04-12

**Authors:** Alexandre Adam, Damien Mertz

**Affiliations:** Institut de Physique et Chimie des Matériaux de Strasbourg (IPCMS), UMR-7504 CNRS-Université de Strasbourg, 23 Rue du Lœss, BP 34 67034 Strasbourg, France

**Keywords:** iron oxide@mesoporous silica, core-shell nanomaterials, magnetic resonance imaging, magnetic hyperthermia, near-infrared light photothermia, drug delivery

## Abstract

The design of core-shell nanocomposites composed of an iron oxide core and a silica shell offers promising applications in the nanomedicine field, especially for developing efficient theranostic systems which may be useful for cancer treatments. This review article addresses the different ways to build iron oxide@silica core-shell nanoparticles and it reviews their properties and developments for hyperthermia therapies (magnetically or light-induced), combined with drug delivery and MRI imaging. It also highlights the various challenges encountered, such as the issues associated with in vivo injection in terms of NP–cell interactions or the control of the heat dissipation from the core of the NP to the external environment at the macro or nanoscale.

## 1. Introduction

Nanomedicine’s main goal is to take advantage of nanotechnologies in order to develop new therapies against diseases such as cancers. One of the main challenges is to produce nanoplatforms able to combine diagnostic and therapeutic properties that are adapted to each disease and each patient. Actual progresses in the synthesis of complex nanoplatforms allow to produce multifunctional nanoparticles (NPs) which pave the way for new opportunities in the field of oncology. This multifunctionality can be achieved by synthesizing NPs with multiple components. In this frame, core-shell NPs are particularly appealing as they allow us to combine the properties of the core and the surrounding coating (the shell). A wide variety of nanomaterials have been developed that could allow us to produce core-shell NPs. They are based on liposomes, lipids, or polymers but also inorganic NPs made of metal oxides, gold, silver, or silica [1,2,3,4].

Among promising nanomaterials, ferrite-based NPs are particularly interesting as their properties make them useful as contrast agents for magnetic resonance imaging (MRI) but also as therapeutic agents by applying an appropriate stimulus (magnetic field, light) so they can generate localized heating. Iron oxides (IO) and especially magnetite, which is the simplest example of ferrite ((Fe^3+^)[Fe^2+^,Fe^3+^]O_4_), are particularly adapted and attractive as they combine suitable magnetic properties, biocompatibility, and biodegradability [5]. These materials show very low toxicity, and IO-based nanoformulations have already been approved for medical imaging applications, such as T_2_ contrast agents for MRI. To ensure good colloidal stability, control the aggregation state, and have a good biodistribution, these IO NPs are generally functionalized on their surface by organic ligands [6] such as polymer chains [7], polyethylene glycol (PEG) [8], or dendrimers [9]. Upon the application of an appropriate alternating magnetic field (AMF), it is possible to generate heat from these NPs. This heat could be used to develop efficient localized therapies based on magnetic hyperthermia [10]. Nanotherm^®^, developed by MagForce Nanotech AG, is the first therapeutic product based on this effect using NPs to be authorized in Europe to treat brain tumors [11]. A wide range of perspectives is, therefore, emerging for nanoformulations based on IO NPs. Currently, one of the main limitations of magnetic hyperthermia treatment comes from the relatively low heating power of existing products, which require an injection of large quantities of NPs (ca. 20–30 mg iron oxide per cm^3^ tumor in average) [12,13]. One of the challenges is, therefore, to produce very effective NPs to significantly reduce the doses administered and thus the potential side effects.

However, this research field still needs to overcome several challenges such as the improvement of the colloidal stability in biological media and in vivo models, the functionalization of the surface with complementary treatment/imaging functions, and the limitation of the NPs’ toxicity. That is why, in order to develop a new generation of theranostic materials, it seems promising to combine properties of IO with other materials to bring new functionalities to overcome these challenges.

In association with IO, the use of porous or non-porous silica in magneto and photoresponsive systems is emerging. The addition of silica around IO cores offers the opportunity to design new smart core-shell NPs. The porosity and the high specific surface area of the silica can be advantageously used for the efficient loading of a wide range of molecules. Pores can be designed to charge drugs, peptides, or proteins. A silica coating would also significantly improve the biocompatibility of a core-shell NP, making it possible to use many materials for biomedical applications. Silica is a biocompatible material that is also biodegradable but over longer times than IO. It is indeed an FDA-approved material that is, for example, used in the form of bioactive glass with other elements (Ca, Na, P) for bone reconstruction [14,15]. Finally, silica surface chemistry is highly versatile and can be modified for effective targeting and “on-demand” drug release. To date, only one nanocomposite formulation is involved in clinical trials and is based on silica-coated gold nanoparticles for photothermal ablation applications, Aurolase^®^ by Nanospectra Biosciences.

Therefore, the development of silica-coated nanocomposites having a responsive inorganic core would open the way toward a new class of biomedical materials combining the properties of the two materials, thus leading to marketable multimodal nanoplatforms.

**Figure 1:**
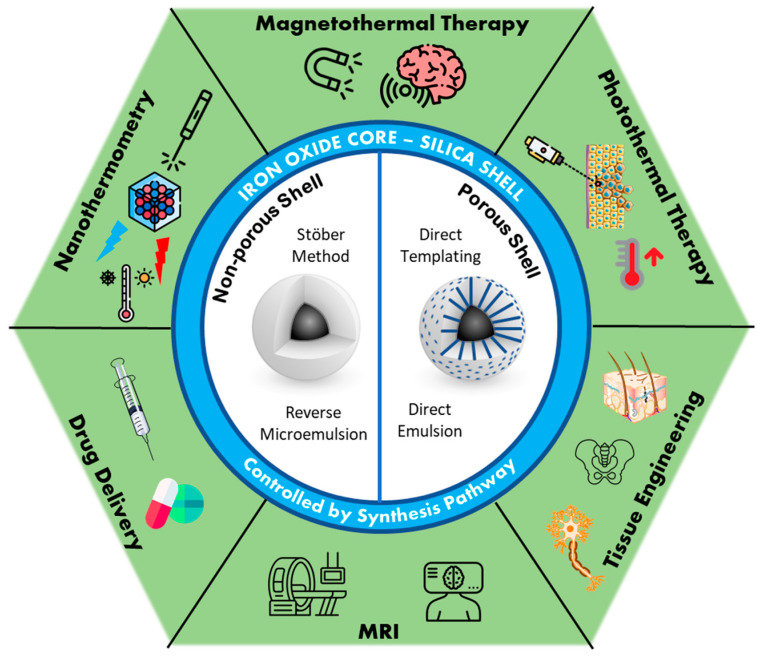
Scheme illustrating the strategies for the design of iron oxide core @silica shell nanomaterials and their potential application for magneto and photothermal therapy hyperthermia, MRI, tissue engineering, nanothermometry, or drug delivery.

**Figure 2:**
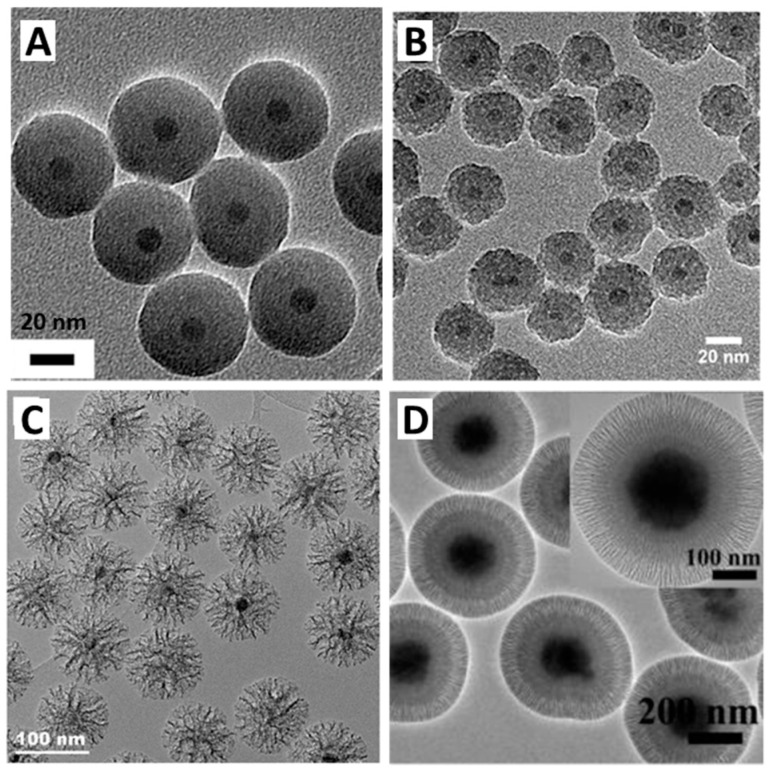
TEM images of iron oxide core@silica shell NPs from the literature with different morphologies of the silica obtained by different synthetic pathways. Non-porous silica (**A**) [16], mesoporous silica with small pores (around 2.5 nm) (**B**) [17], mesoporous silica with larger pores (about 10 nm) (**C**) [18], and a silica shell made of two layers with an interior non-porous silica coating, above which sits a mesoporous silica shell with radially-oriented large pores (10 nm) (**D**) [19]. Reprinted (adapted) with permissions from Refs. [16,17,18,19].

In this article, we review different strategies that have been developed in recent years to deposit a silica shell around iron oxide core materials and we describe their potential application for MRI, magnetic hyperthermia, NIR light–photothermia, or drug delivery (Figure 1). In the first section (part II), after reviewing the properties and synthesis pathways of superparamagnetic iron oxide nanoparticles and mesoporous silica individually, a focus is made on the synthesis strategies used to cover IO NPs with non-porous and porous silica shells. Some examples of the nanocomposites obtained in the literature are represented in Figure 2. Core-shell iron oxide@silica NPs are considered for theranostic applications. We detail, in the second section (part III), their physical properties, such as proton magnetic relaxation for MRI and the mechanisms by which they produce heat upon alternating magnetic field or NIR light. Special attention is given to local temperature understanding and measurement by using nanothermometers. Furthermore, in this section, we also report examples of drug delivery systems based on core-shell NPs. In the last section (part IV), to demonstrate the potential of such nanomaterials for nanomedicine, some biological applications of core-shell NPs, either as circulating nanoobjects or as components of smart activated hydrogel composite scaffolds, are presented.

## 2. Iron Oxide Core@Silica Shell NPs

### 2.1. Iron Oxide Generalities

#### 2.1.1. Crystal Structure

Iron oxide compounds are very common materials that are widespread on Earth and can be found in eight different crystallized structures [20]. Among these, iron oxide spinel phases, magnetite Fe_3_O_4_, and maghemite γ-Fe_2_O_3_ are the most promising, as they show ferrimagnetic behavior as bulk and superparamagnetism at the nanometric scale.

**Figure 3:**
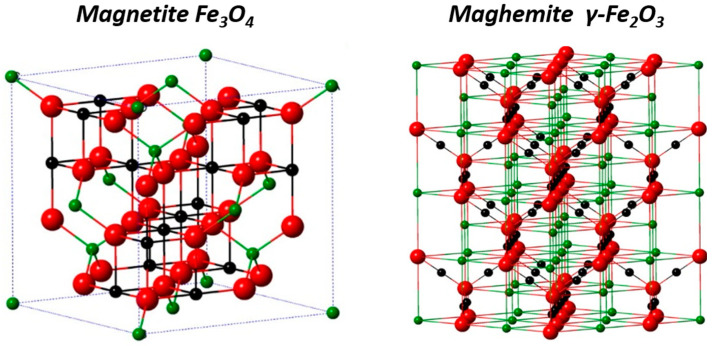
Crystalline structure and crystallographic data of the magnetite (left), and maghemite (right) (the black sphere is Fe^2+^, the green sphere is Fe^3+^, and the red sphere is O^2−^) [21]. Adapted with permission from [21] under the terms of the CC BY license.

Magnetite Fe_3_O_4_ is an inverse spinel structure AB_2_O_4_ with divalent cations in B sites. The crystalline unit cell is based on 32 oxygen anions forming a face-centered cubic structure which has 64 tetrahedral and 32 octahedral sites. ⅛ of the tetrahedral sites are occupied by Fe^3+^ cations (A sites) and ½ of the octahedral sites are occupied by Fe^2+^ and Fe^3+^ (B sites), leading to the formula (Fe^3+^)_A_[Fe^2+^ Fe^3+^]_B_(O^2−^)_4_. Maghemite is an oxidized form of magnetite with a cubic structure. To compensate for the oxidation of Fe^2+^ into Fe^3+^, vacancies appear in the octahedral sites in order to have an overall neutral structure and it can be written as (Fe^3+^)[(Fe^3+^)_5/3_□_1/3_]O_4_ which is equivalent to Fe_2_O_3_ (Figure 3).

#### 2.1.2. Superparamagnetism

**Figure 4:**
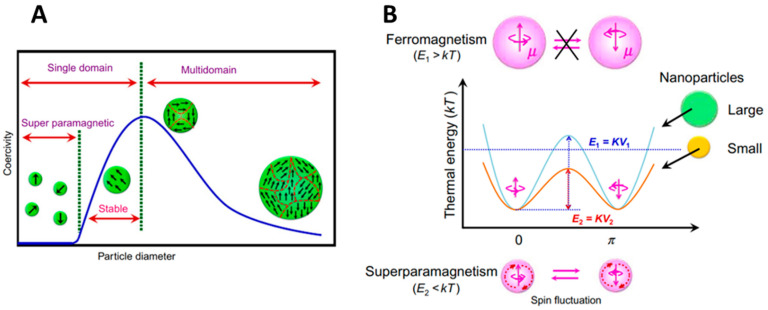
(**A**) Size-reliant domain structures from superparamagnetism to ferri or ferromagnetism. (**B**) Energy diagram of single-domain NPs corresponding to different macrospin alignment ferromagnetism in bigger particles (up) and superparamagnetism in NP (down) [22]. Reprinted with permission from [22].

Historically, magnetite was considered a ferromagnetic material until Louis Néel discovered ferrimagnetism and changed its classification. As a bulk material, magnetite is ferrimagnetic which means that inside the material the magnetic moments are antiparallelly distributed but with different amplitudes, resulting in a remaining spontaneous magnetization. The bulk magnetite is composed of magnetic domains in which the magnetization has a uniform direction. By decreasing the size of the material, the number of domains decreases until a critical diameter D_c_ where individual objects are composed of a unique magnetic domain (Figure 4A). Thus, the total magnetization of the NP is a single giant magnetic moment coming from the sum of all magnetic atom’s moments and is represented as one macrospin. D_c_ is around 30–40 nm for IO NPs.

The energy of a single-domain NP is generally dependent on the magnetization direction with respect to the easy axis. The macrospin has two equilibria at antiparallel directions along the magnetization easy axis. To switch from one direction to the other (Néel relaxation), it is necessary to overcome an energy barrier KV_p_ (anisotropy energy) with K, the magnetic anisotropy constant, and V_p_, the volume of the particle (Figure 4B) [16]. Thus, in the single-domain range, at any given temperature, there is a critical size below which thermal agitation is sufficient to cross this barrier and rotate the NP magnetization. Therefore, when no magnetic field is applied, the orientations of the macrospins are distributed randomly and the sum of the macrospins of the NPs is zero. When an external magnetic field is applied, the NPs tend to align in the field direction, resulting in a net magnetization that disappears when the magnetic field is switched off. This phenomenon is referred to as superparamagnetism. For IO NPs, the critical size for blocked single-domain and superparamagnetic behavior is about 20 nm, depending on the composition and shape. Inversely, for a given size where NPs are superparamagnetic (k_B_T >> KV), by decreasing the temperature, there is a given temperature for which the energy is lower than the anisotropy energy, which is called the blocking temperature. In this review, the focus is made on magnetite and maghemite but it can be noted that the doping of ferrites, in particular with rare-earth elements, can significantly modify the spinel structure and thus the magnetic properties, such as anisotropy of the crystal lattice and magnetization [23,24,25]. It is also possible to significantly enhance the luminescence properties by doping these ferrites with other metals, such as gadolinium or erbium [26,27,28,29].

#### 2.1.3. Iron Oxide Synthesis

Synthesis routes for IO NPs are today very diversified and allow to obtain high-quality magnetite NPs. Three main routes can be distinguished: physical, chemical, and biological. Physical methods include chemical vapor deposition [30], pulsed laser ablation [31,32,33,34], laser pyrolysis [35,36], and mechanochemical [37,38] techniques. They can be easy to perform but the morphology and size of the NPs are more difficult to control. It also requires expensive equipment. Wet chemical routes represent the more robust way of synthesizing IO NPs and the more mature area. The methods are very diverse and by fine tuning the reaction conditions, it is possible to obtain narrow size distributions and precise composition. Biological routes deal with the use of bacteria and microorganisms to synthesize the NPs. They are usually of excellent quality but quite time-consuming and complicated to handle after synthesis [39,40]. We will briefly review the chemical methods, as they are the most reported routes to produce IO NPs and they have low production costs and high yield. Among these methods, the most famous include co-precipitation, polyol or hydrothermal methods, microemulsion, and thermal decomposition.

##### Co-Precipitation Method

Co-precipitation from aqueous solutions is the most common method to synthesize commercial IO NPs due to the low cost of the reactant, the very short reaction time, and its ease to upscale for industrial production. It consists of the precipitation of Fe(II) and Fe(III) salt solutions by the addition of an aqueous basic solution (dissolved sodium hydroxide or ammonia, for example). As a result, a black dispersion of IO NPs is produced whose shape and size depend on different reaction parameters. pH and ionic strength are crucial parameters to control the size of the particles. In the 1980s, Massart was one of the first to report the alkaline synthesis of such IO NPs by using ferric and ferrous chloride salts [41]. The high surface-to-volume ratio usually pushed the NPs to aggregate to decrease the surface energy. That is why surfactants [42], polysaccharides [43], protein, polyelectrolytes can be used as dispersing agents to stabilize the obtained NPs [44,45]. Many synthesis parameters have to be taken into account and have an impact on the properties of the resulting IO NPs. Indeed, different parameters such as the counterions, the concentration of cations, the choice of the base, the reaction temperature, the stirring rate influence the size, the phase (the composition), and the shape which lead to the variability of the physico-chemical properties [46,47,48,49,50]. Even if tremendous efforts have been performed to improve the obtained NPs and the reproducibility of the syntheses, the resulting IO NPs present a quite large polydispersity and limited shape control. To overcome these limitations, other chemical processes have been developed.

##### Polyol Method

The shape and size control by the polyol method improved compared to the classical co-precipitation. Here, the solvents used, polyols or polyethylene glycol, have a high dielectric constant and a high boiling point, which offer the possibility to operate on a broad range of temperatures. Polyols act as the solvent (they can dissolve inorganic compounds) but also have the function of both reducing and stabilizing agents. Thus, they have a major role in the control of the NP growth and preventing their aggregation. Shape, size, and yield of the reaction depend on the choice of polyols, the ratio of iron salts, concentration, and the used as other additives [51,52].

##### Hydrothermal Method

The hydrothermal method is after the co-precipitation method, the second most developed route for the synthesis of IO NPs [53]. The reaction occurs in an aqueous solution in a closed reactor or autoclave. By heating above the standard boiling point of the solvent, the pressure of the liquid increases, and it is possible to reach high pressure (>100 bar) and high temperature (>200 °C). Compared to co-precipitation which uses equivalent reactants, the size and shape control of the IO nanocrystals improved. However, more energy is needed, and the reaction time is relatively long. So, to be more efficient, the microwave-assisted hydrothermal method has been implemented [54,55]. A complete study of the different synthesis parameters has been reported by Hao et al. to verify the influence of precursors, time, and temperature on the obtained IO NPs [56].

##### Thermal Decomposition

**Figure 5:**
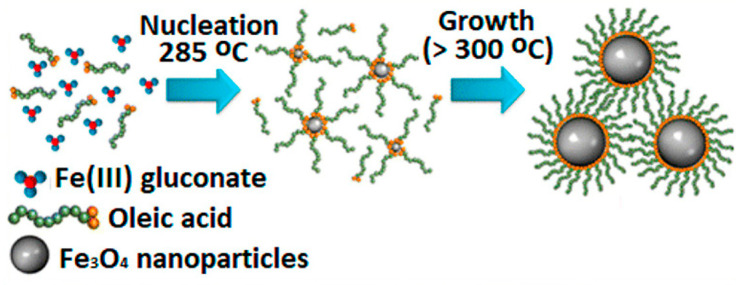
An example of the thermal decomposition of iron(III) gluconate to synthesize superparamagnetic Fe_3_O_4_ nanoparticles [57]. Reprinted (adapted) with permission from Patsula et al. [57,58].

This process consists of heating at high temperature a metallic complex in order to induce its decomposition. Organic solvents with high boiling points are needed, as well as surfactants to stabilize the formed NPs. When decomposing, the metal ions from the complex crystallize which forms nuclei that will further grow to form the NPs. This leads to highly monodisperse IO NPs that are very well stabilized in organic solvents thanks to the coating of surfactant (Figure 5). For example, oleic acid allows good stability in chloroform or THF. This method is appropriate to synthesize high-quality IO NPs with a narrow size distribution, as the nucleation step and the growth step can be easily separated.

Few iron precursors are commonly used to produce IO NPs, especially magnetite NPs. Among them, Fe(acac)n [59,60], iron oleate [61], carbonyls Fe(CO)x [62,63], or iron stearate [64,65] are the most represented. In short, this method offers the best control of the synthesis parameters and allows the production of IO NPs with precise size and shape. At the end of the reaction, the stabilization by the surfactant leads to good stability in organic solvents which will be advantageously used to synthesize the mesoporous silica shell.

### 2.2. Silica and Mesoporous Silica Nanomaterials

**Figure 6:**
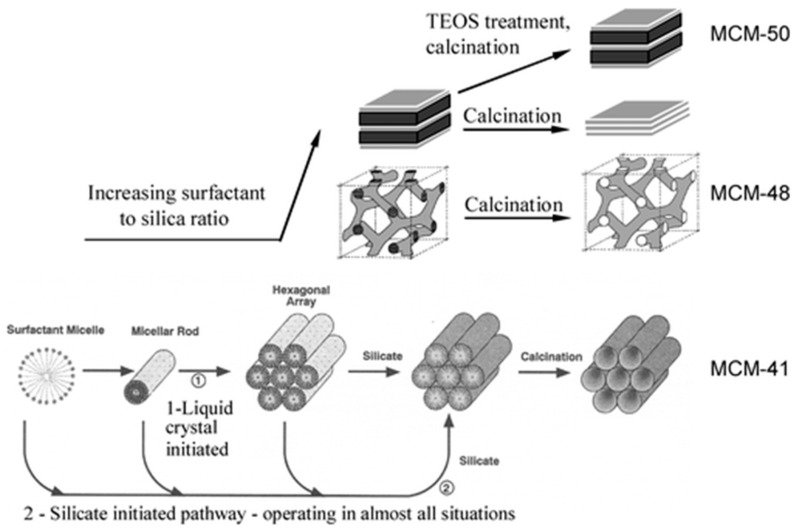
The proposed LCT pathways (lower half) and its other structures by increasing surfactant to silica ratio (upper half) [70]. Reprinted with permission from [70].

In the early 1990s, two groups of scientists from Mobil Oil Corporation [66] and Kuroda’s group [67,68], reported independently the synthesis of mesoporous silica using surfactants as templating agents. The production of these structures, in particular, the famous MCM-41 and all the M41S-family [69], was a major discovery and it immediately attracted attention from the materials science community. Today, it is a major field of research due to the numerous applications and possibilities these materials are offering. The products obtained exhibited high surface specific areas whose pore dimensions were easily tunable between 2 and 10 nm by adding co-solvents or changing the surfactant. The regularity of the pores’ arrangement in the structures was demonstrated by well-defined X-ray diffraction patterns from this amorphous material that is silica. The formation mechanism of these materials was called liquid crystal templating (LCT) by analogy with liquid crystal phases involving mixtures of water and alkyltrimethylammonium salt surfactants. Figure 6 shows the first supposed liquid crystal-initiated synthesis pathway [69].

However, by keeping all the synthesis conditions constant but modifying the amount of silica precursor in the reactive mixture (change of surfactant/silica precursor molar ratio), they formed other structures known as MCM-48 (cubic) and MCM-50 (lamellar), for example. Thus, these experiments invalidated the hypothesis of a preformed liquid crystalline phase prior to the formation of the silica network [70]. It is the interaction of the silicate species (anionic) formed during the reaction with the surfactant micelles (cationic) which leads to the formation of the different silica phases following a mechanism named cooperative self-assembly. Silica oligomer anions exchange with HO^−^ and Br^−^ (in the case of cetyltrimethylammonium bromide (CTAB)) to form inorganic–organic aggregates whose structures are different from the initial surfactant micelles. By strongly screening the electrostatic repulsion, these aggregates can self-assemble into an organized mesophase [71]. Since then, a broad variety of materials have been developed inspired by the synthesis strategy of the M41S family, including monolithic, micro-sized, and nano-sized materials.

The development of nanometric MCM-41 particles was reported only a few years after the original paper by Mobil Oil Corp [72,73,74]. Today, a wide majority of the mesoporous (MS) NPs are synthesized by using CTAB surfactant and tetraethyl orthosilicate (TEOS) as precursors. The silica condensation is catalyzed in basic conditions which usually yields hexagonally packed mesopores around 3 nm. Similar to the previous materials, the porosity can be tuned either by the addition of co-solvents in order to swell the surfactant template pores or by the use of surfactants with longer hydrophobic chains [75,76,77]. Mesitylene is a well-known swelling agent used to increase the pore diameter [78]. With the addition of ethanol and/or ethyl acetate in water and varying the volume ratio of ethanol/ethyl acetate, it was possible to modify the pore structure from parallel to radial and to worm-like [79].

The nature of the surfactant is also crucial to determine the pore structure. After hydrolysis of the silica precursors, the resulting silicate species interact with the positively charged micelles of cetyltrimethylammonium surfactant (CTA^+^). These interactions change the shape of the micelles around which the silica is forming. CTA^+^ is commercially available as a salt and thus associated with a counterion. Zhang et al. showed that the counterion, by competing with the interaction between the silicate and the micelles, also directs the shape of the pores [80]. The change of counterion from bromide (Br^−^) to tosylate (Tos^−^) leads to a dramatic change of pore morphology from raspberry-like [81] to stellate [82,83]. Furthermore, by adding anionic polymers, such as poly(acrylic acid) (PAA), an organic complex having a specific ordered structure is formed. This resulted in an original silica-based mesostructure having a bimodal porosity distribution [84]. Wang et al. also showed that by increasing the concentration of PAA, the diameter of the spherical nanoparticles increases as well as the pore size [85]. The use of nonionic amphiphilic block copolymers instead of cationic surfactants as directing agents also allows the synthesis of silica materials with different pore sizes. Diblock or triblock copolymers made of commercially available poly(ethylene oxide) (PEO), poly(propylene oxide) (PPO), and polyolefin or polystyrene are mostly used [86,87,88,89,90,91]. These have the advantage that their ordering properties can be very finely tuned by adjusting the copolymer architecture, the ratio and composition of hydrophobic/hydrophilic parts, the molecular weight, and the solvent used. In the last two decades, extensive research about MS materials led to their diversification into a variety of research fields.

MS NPs are particularly popular for biomedical applications due to their controllable particle size [92], tunable pore size and distribution [75], large surface area and high pore volume, versatile surface modifications [93], and good biocompatibility [94]. In 2001, Vallet-Regi et al. [95] reported the first use of MCM-41 as a drug delivery system. Since then, the use of MS NPs in nanomedicine has been the object of intense research, as proven by the large number of scientific reviews about this topic [96]. The remarkable advantages of large specific surface and pore volume, as well as large pore diameter, are very well-suited for the transport of a large number of small drug molecules and bulky biomolecules, DNA, RNA, peptides, and proteins [97]. A major challenge to overcome in the future to produce safe clinical products is the degradability of the MS NPs. MS NPs have been shown to degrade in biological media into silicic acid Si(OH)_4_, which is nontoxic and water-soluble [98]. However, because of its good stability, the rate of degradation is relatively slow (at least a few weeks to months) and thus the retention in the body during this period can cause adverse effects. To have an efficient circulation half-time, the size of the NP is a fundamental parameter. Prolonged retention within the blood circulation and mitigated renal clearance can be obtained by optimizing the size [99]. Indeed, particles in the micrometric range could be easily metabolized by active phagocytosis via the mononuclear phagocyte system. On the other hand, very small NPs (<5 nm) which are capable to pass easily through cell membranes are quickly eliminated via renal clearance [100].

Today, various research works in the nanomedicine field aim at developing efficient smart nanocarriers. This refers to stimuli-responsive drug delivery systems which will vehicle the therapeutics in the body to the required site in the safest and most efficient manner. The first generation of cargo nanocarriers as lipid NPs or liposomes usually show adverse effects coming from their fragility, nonspecific biodistribution, limited targeting, and uncontrollable drug release [101]. On-demand drug delivery systems based on MS NPs have shown very promising results by reducing premature unwanted leakage [102,103,104]. This new step toward smart nanocarriers was possible by the addition of responsive nanocaps to block the pores, known as gatekeepers [105]. Today, a variety of gatekeepers have been developed to trigger the release when applying a defined stimulus. Internal stimuli deal with the use of a pH change, redox potential, or enzyme to stimulate the drug release. External or remote stimuli are based on the application of light, magnetic field, or temperature change. Internal triggers are directly related to the chemical and biological environment of the target site in the body. For instance, (endo)lysosomal pH is decreased between 5.5 to 4.5, protease concentration increases, and ionic force changes [106]. pH in cancer tumors is also known to be lower than physiological pH [107]. All these phenomena can be advantageously used to design smart nanocarriers.

In summary, MS materials are quite easy to synthesize in mild conditions and controlled environments and have remarkable properties. Their combination with other materials would thus lead to a new generation of nano-architectures. These nanocomposites would have new modalities combining properties of each material and thus new imaging and therapy properties; i.e., releasing heat or triggering drug delivery, in response to external stimuli, or bearing imaging agents, while having key physicochemical features such as colloidal stability, non-toxicity, and versatile functionalization. Surface properties are brought by the silica which can be coated around various activated material cores such as iron oxide materials.

### 2.3. Non-Porous Silica Shell Coating around Iron Oxide

Once IO NPs have been synthesized, the concept is to use them as cores for silica shell coating. This would lead to their complete coating with a non-porous or mesoporous silica shell. This shell has an important impact on the properties of the newly formed nanocomposite. In terms of stability, the silica protects the IO core from dissolution while bringing very good colloidal stability in various solvents and, especially in an aqueous buffer thanks to their negatively charged surface. It also prevents any aggregation between the magnetic cores originating from their dipolar interactions or/and hydrophobic surface chemistry. IO NPs synthesized by thermal decomposition are coated with ligands, such as oleic acid, and are thus stable in organic solvents, such as chloroform or tetrahydrofuran. Thus, they need to be transferred into an aqueous medium so that the silicate species can polymerize around the IO cores.

#### 2.3.1. Coating by Stöber Sol-Gel Method

Probably the most direct and facile method is to coat IO NPs with non-porous silica using a surfactant-free, low-tech, and low-cost process [108]. This facile and famous route is known as the Stöber, Fink, and Bohn process and is among the first method used to coat inorganic NPs with silica [109]. The synthesis involves the dispersion of IO NPs in an ethanolic solution, followed by the hydrolysis/condensation of the silica precursor (commonly TEOS) onto the surface of IO NPs [108,110,111]. The reaction is usually catalyzed by ammonia. The generation of silica from silica precursors follows a mechanism named hydrolysis/condensation. First, in the presence of ammonia, in an ethanol solution, the alkoxide (Si-O-R) hydrolyzes so that alkoxy groups (O-R) are replaced by silanol (Si-OH) monomers (Equation (1)). Then, the silanol monomers condense to form siloxane bonds (Si-O-Si). This generates branched siloxane clusters which will further react together to form silica nuclei and trigger the growth of silica nanoparticles (Equation (2)). Silanol monomers can also react with a non-hydrolyzed silica precursor (TEOS) via direct condensation between silanol and alkoxy groups which will participate in silica network formation (Equation (3)) [112].

Si(OEt)_4_ + xH_2_O ⇌ Si(OEt)_4−x_(OH)_x_ + x EtOH (1)

2 Si(OEt)_4−x_(OH)_x_ ⇌ (EtO)_8−2x_(Si-O-Si)(OH)_2x−2_ + H_2_O (2)

Si(OEt)_4_ + Si(OEt)_4−x_(OH)_x_ ⇌ (EtO)_7−x_(Si-O-Si)_1_(OH)_x−1_ + EtOH (3)

Hydrolysis of TEOS occurs via nucleophilic substitution of ethoxy groups for OH groups. The addition of ammonia in the mixture increases the concentration of HO^−^ ions which are a lot more efficient nucleophiles compared to H_2_O. Moreover, when bulky groups (OEt) are removed for OH, the hydrolysis rate increases by lowering the steric hindrance around Si atoms. For its part, condensation between neighbor silanols also involves a nucleophilic attack. However, in these basic conditions, condensation is much faster than hydrolysis because the silanol groups, which are the actors of the nucleophilic substitution, are deprotonated more easily than water molecules. Si atom thus become more electrophilic (more electropositive), which is favorable for the attack. By doing so, we understand that silanols preferentially condense into large branched siloxane networks rather than onto small oligomers. This leads to a relatively good size control by favoring growth over nucleation. The kinetic balance between hydrolysis and condensation is fundamental to having a monodisperse NP distribution. Silica shells with thicknesses between 2 to 100 nm can be obtained by controlling the reaction parameters such as coating time, reactant concentrations, and the presence of a catalyst or other precursors than TEOS [113]. This synthesis method is usually more adapted to IO NPs that are not coated with surfactants. A major limitation is usually the lack of control and the polydispersity of the silica thickness. To circumvent this issue, reverse microemulsion routes were developed.

#### 2.3.2. Non-Porous Silica Coating by Reverse Microemulsion Process

**Figure 7:**
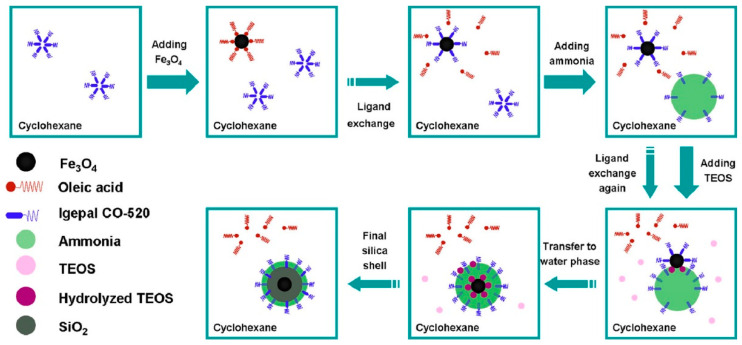
Mechanism of coating of IO NPs with silica via microemulsion [16]. Reprinted with permission from [16].

Microemulsion synthesis is a form of the seed-mediated growth process. The IO cores are previously synthesized and act as seeds for silica shell growth. This process requires to have IO NPs capped with an organic ligand, such as classical oleic acid, and be dispersed in a non-water-soluble organic solvent. Hence, reverse emulsion (water in oil) can be set up to obtain homogenous non-porous silica shells. The dispersion is mixed with a surfactant solution and aqueous ammonia is added in order to form an emulsion [114]. By further addition of silica precursors and its subsequent partial hydrolysis, the IO NPs enter the ammonia droplets where the silica condenses around the cores [16]. Uniform coating of a single NP can thus be obtained by controlling the ratio between the number of droplets and the number of IO NPs. Modification of the synthesis parameters leads to modulated silica thickness (Figure 7). Thus, each IO NP is found within a droplet which constitutes a microreactor. Compared to the Stöber process, this method results in a much more controlled non-porous silica shell.

In order to develop mesoporous silica shells, other methods have been developed. They involve the use of surfactants whose organization in the presence of a silica precursor creates periodically distributed hybrid inorganic/surfactant domains. After the removal of the surfactant, this results in mesopores in the structure.

### 2.4. Porous Silica Shell Coating around Iron Oxide

#### 2.4.1. Mesoporous Silica on IO NPs via Direct Templating

The first method is equivalent to the synthesis of MCM-41 using surfactant in an aqueous solution but in presence of IO NPs. Duguet and coworkers [115] developed this method to coat IO NPs synthesized by coprecipitation. Surfactants are very useful molecules in many areas thanks to their specific structures. They are composed of a polar head and a non-polar tail, which allow them to assemble at the water/oil interface. The most famous and widespread surfactants are the ones based on quaternary ammonium, in particular cetyltrimethyl ammonium bromide (CTAB). In an aqueous solution, the hydrophobic parts assemble themselves to form micelles with hydrophobic cores. However, the hydrolyzed silica precursor is water-soluble, and thus silica oligomers will condense around the ammonium polar head. This complex interaction between the surfactant and silica oligomers leads to the formation of cylindrical hybrid micelles whose walls solidify as the silica forms. This is why CTAB plays the role of templating agent. However, it can be difficult for the silica-CTAB complex to anchor directly to uncoated IO. Deng et al. [116] prepared IO nanoclusters by a solvothermal method which are thus not prone to coating with surfactant. They proposed to first synthesize a sublayer of non-porous silica via the Stöber method, which then allowed a good coverage with CTAB, and subsequent mesoporous silica.

#### 2.4.2. Mesoporous Silica Coating on IO NPs through Direct Emulsion

**Figure 8:**
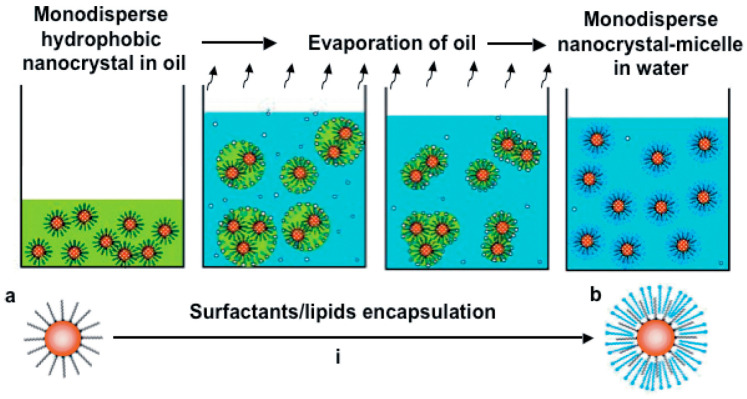
Representation of the formation of water-dispersible IO NPs through surfactant encapsulation [121]. Reprinted (adapted) with permission from [121].

IO NPs that are synthesized with the best shape and size control method, e.g., by thermal decomposition, are stabilized with organic ligands which make them dispersible in apolar organic solvents. To coat these NPs, new methods have been established and imply passing through an emulsion step. Direct emulsions use surfactants in order to stabilize in water the non-water dispersible IO NPs. IO NPs capped with an organic ligand and dispersed in a non-water-miscible organic solvent are needed. Briefly, the organic-solvent IO NPs suspension is added to an aqueous solution of surfactant (such as CTAB) and a base. Under strong agitation, the organic solvent mixes into the aqueous phase to form an oil-in-water microemulsion which is stabilized by the surfactant [117]. Then, the organic solvent is removed by controlled evaporation. By slowly heating and removing the organic solvent, the surfactant molecules will interact with the coating molecules of IO NPs through hydrophobic interactions between the alkyl chains. This forms a thermodynamically interdigitated bilayer structure of surfactant around the NPs. In order to stabilize them in an aqueous phase as shown in Figure 8. Hyeon et al. [118] reported in 2006 the first encapsulation of IO NPs via this method, using CTAB as the stabilizing and templating agent. The hydrophobic properties of the IO NPs ensure an optimal distribution of the magnetic cores into the micellar phase [119,120]. As the IO NPs that we used are coated with oleic acid, this is the preferred method for the deposition of the silica shell. This results in very monodisperse and homogeneous IO@MS NPs.

The control of the shell thickness growth is one of the most important features. In the case of this synthesis method, the ratio of CTAB surfactant and TEOS is primordial.

Ye et al. [117] showed that a large excess of CTAB during the reaction results in the formation of mesoporous silica without an IO core. As detailed above for the synthesis of mesoporous silica NPs, the use of a swelling agent such as mesitylene or the increase in the length of the alkyl chain of the surfactant allows increasing the width of the mesopores. In an original method, Yang et al. [19] coated IO nanoclusters via a biphasic oil-in-water coating strategy in which the oil phase enables a swelling of the pores resulting in large dendritic open pores. In another method from our group, the temperature of the sol–gel was found to be a crucial parameter to orient pore morphology either toward ST or WLMS [18].

It is important to note that the production of periodic mesoporous organosilica (PMO) nanoparticles obtained from the condensation of organosilanes is a class of hybrid organic–inorganic mesoporous material developed for a decade that holds great promise for many applications. Thanks to the diversity of the organic fragments, it is possible to generate pore walls bearing almost any chemical group. In particular, for biomedical applications, the introduction of organic groups greatly modifies the hydrophobicity, the interaction with loaded drugs, and the biodegradability of these NPs [122,123,124,125,126]. For example, the insertion of disulfide functions brought very good biodegradability to the nanoobjects [127,128,129].

Hence, the combination of IO with MS gives rise to core-shell nanocomposites IO@MS which have unique features. Their main properties are described in the following paragraphs.

## 3. Physical Properties of IO@MS as Theranostic Agents

As detailed above, the main interest to design IO@MS for biomedical applications is to combine the therapy and diagnosis abilities of each compound. Thus, IO@MS can be used as MRI contrast agents thanks to the IO core but also as therapy vehicles with MHT or phototherapy treatments as well as drug delivery carriers (chemotherapy) thanks to the porous MS shell.

### 3.1. IO@MS as MRI Contrast Agents

#### 3.1.1. MRI and Contrast Agent Principles

**Figure 9:**
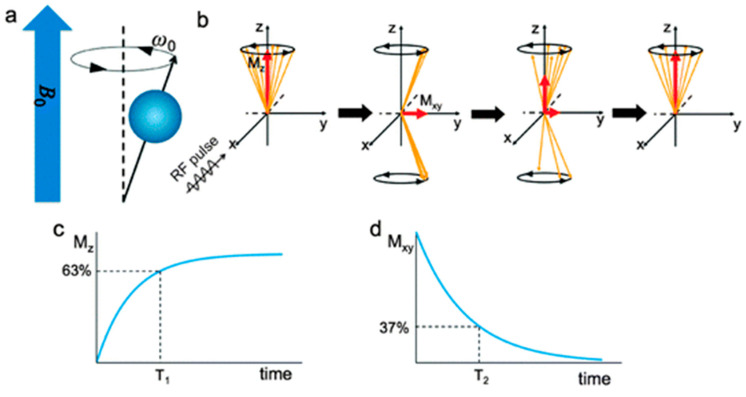
Principles of magnetic resonance imaging (MRI). (**a**) In a magnetic field, the hydrogen nuclear spins align with (parallel) or against (antiparallel) the external magnetic field. (**b**) Irradiation of resonant RF results in a decrease in longitudinal magnetization (*M_z_*) and the generation of transverse magnetization (*M_xy_*). Subsequently, the nuclear spins return to their initial state, referred to as relaxation. (**c**,**d**) *T*_1_ is the time required for longitudinal magnetization to recover to 63% of its equilibrium (**c**) and *T*_2_ is the time required for transverse magnetization to drop to 37% of its initial magnitude (**d**) [131]. Reprinted with permission from [131].

Magnetic resonance imaging (MRI) is today a very common non-invasive medical imaging technique used for the diagnosis and follow-up of many diseases, in particular cancers, whose solid tumors can be detected. MRI is based on the physical phenomenon of nuclear magnetic resonance of hydrogen atoms. These atoms are particularly abundant in the human body; especially in water and fat and represent about 63% of its mass. When the protons are exposed to a strong static magnetic field B_0_ (along the *z*-axis), their spin aligns (parallel or antiparallel) to the direction of this field, and they are processed under the Larmor frequency ω_0_. Parallel orientation is the lower state thus it is slightly preferred. This results in a net magnetization vector M_z_ parallel to B_0_. Then, a resonant radiofrequency pulse (RF) with a resonance frequency equal to the Larmor frequency is applied perpendicularly to B_0_. This irradiation is absorbed by the protons (nuclei of hydrogen atoms) whose spins jump from the parallel state to the higher level of antiparallel state and secondly, the spins are “whipped” to precess in phase. The effect of all this is that the net magnetization M_z_ flips 90° from the *z*-axis to the transverse plane [130]. The magnetic moment of the protons is a vector with two contributions: M_z_, the longitudinal magnetization parallel to B_0_ and M_xy_, the transverse magnetization. As RF stops, the nuclear spins relax and return to their initial equilibrium state. Figure 9a,b summarizes this mechanism. In Figure 9c,d, the two relaxation processes that proceed independently are depicted. First, (i) longitudinal relaxation, which consists of the realignment with B_0_. The characteristic time of this process is T_1_ and corresponds to 63% of the equilibrium value. Second, (ii) transverse relaxation, for which the magnetization component on the transverse plane (M_xy_) returns to zero. T_2_ is the characteristic time and corresponds to a drop of 37% of the initial magnitude on the xy plane. From these relaxation times, it is possible to reconstruct a 3D image of the patient’s tissues.

In the body, the intrinsic contrast is often not sufficient to provide clear images of abnormal tissues, and contrast agents (CAs) can be injected in the patient to modify the relaxation times of protons around them and thus enhance the overall contrast. Among the most common CAs, paramagnetic ions or complexes, such as gadolinium (Gd^3+^) and manganese (Mn^2+^), are used as T_1_ CAs by reducing the longitudinal relaxation time. The locations around these CAs are highlighted and brighter than without CAs. Ferri- or ferromagnetic compounds, such as iron oxide, will induce local magnetic field gradients which shorten the transversal relaxation T_2_ resulting in hypocontrast, i.e., a dark contrast in T_2_-weighted images. The relaxation rate R is equal to the inverse of the relaxation times. For both relaxation processes, R_1_ and R_2_ (in s^−1^) can be calculated from T_1_ and T_2_, respectively. In the presence of a CA, another parameter called relaxivity can be introduced. The relaxivity represents the efficiency of the CA to relax protons. Thus, for longitudinal and transversal relaxivities r_1_ and r_2_ (in mM^−1^·s^−1^), we have:

$$R_{i,obs}=R_{i,0}+r_{i,CA}\cdot C=\frac{1}{T_{i}}+r_{i,CA}\cdot C$$

where $R_{i,obs}$ is the observed relaxation rate, $R_{i,0}$ the relaxation rate of protons in absence of CA, $r_{i,CA}$ the contrast agent relaxivity (mM^−1^·s^−1^), C the concentration of CA (in mM^−1^), and $T_{i}$ the relaxation time.

**Figure 10:**
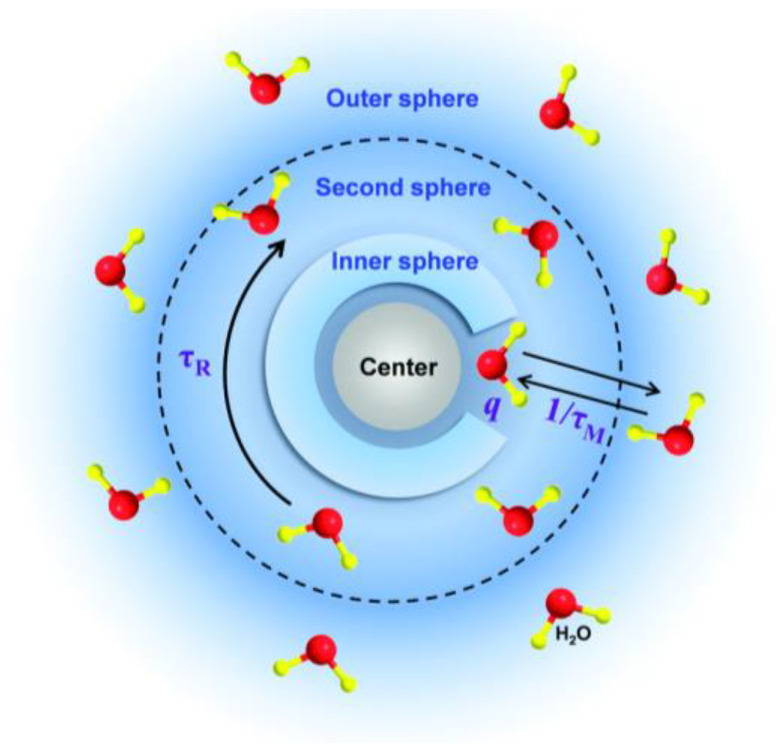
The inner sphere and outer sphere can influence the relaxation rates of MRI CAs [132]. Reprinted with permission from [132].

MRI CAs act in two ways from a molecular point of view The diffusion of water molecules (hence protons) and inner- and outer-relaxation sphere processes need to be considered (Figure 10). Inner sphere relaxation is based on the direct energy transfer between protons and electrons of the first hydration sphere around the CA. It requires close accessibility of water molecules to the CA molecules or NPs and is strongly dependent on the exchange rate of water molecules. Water protons need to be in close proximity to paramagnetic ions to experience fast relaxation. The T_1_-contrasting effect is based on this inner-sphere relaxation. For T_2_-contrasting agents, the dominant contribution comes from the outer-sphere relaxation. This interaction at a greater distance comes from the interaction of protons with the local magnetic field generated by superparamagnetic NPs. The nature of the coating of IO NPs is fundamental as it modifies the interaction with water molecules and thus the contrast produced in MRI.

#### 3.1.2. IO@MS as MRI Contrast Agents

The coating of a non-magnetic silica shell around the IO NPs influences the relaxivities of the nanocomposites. r_1_ and r_2_ values are directly linked to the magnetic properties of the iron oxide core while the silica coating can influence these values as a function of the thickness and the porosity of the coating. Ye et al. [117] studied the influence of the thickness of a mesoporous silica shell around IO NPs on the r_1_ and r_2_ relativities. They showed that IO NPs with a CTAB coating (IO-CTAB) have an r_1_ relaxivity about 10 times higher compared to the same NPs coated with a mesoporous silica shell with small pores between 1 and 3 nm wide. This is attributed to the difference in mobility of water molecules around and inside these coating layers. For IO@MS, the increase in the thickness of the coating also leads to a decrease in r_1_, which is explained by the ability of the MS shell to separate water from the surface of magnetite NPs. Concerning the transversal relaxation (r_2_), which is actually a more relevant mode for MRI than the longitudinal one (r_1_) with IO@MS systems, they showed that transverse relaxivity r_2_ decreases by increasing MS shell thickness, which can be attributed to a weakening of the locally generated magnetic field by IO cores. Pinho et al. [133,134] coated maghemite NPs with non-porous, amorphous silica shells. They also obtained a decrease of r_1_ and r_2_ relaxivities due to the decrease in the inner and outer sphere contributions, respectively. Zhang et al. [135] also demonstrated that for the same thickness, the use of a more hydrophobic silica-based shell decreases r_2_ and r_1_ keeps constant. However, this decrease in r_2_ with a coating of mesoporous silica shell is usually limited, and the more open the pores are, the smaller the impact of the shell is [18,136]. For similar silica thicknesses, Adam and coworkers obtained r_2_ = 307 mM^−1^·s^−1^ with a silica shell having pores around 12 nm wide and r_2_ = 156 mM^−1^·s^−1^ when the pores were 4 nm wide. At last, by using the pores of MS to load paramagnetic gadolinium ions in iron oxide-mesoporous silica core-shell nanocomposites, Gao et al. [137] managed to produce a dual T_1_ and T_2_ contrast agent. They showed that with their construction, by increasing the concentration of NPs, T_1_-weighted MR images become brighter, and T_2_-weighted MR images become darker. Relaxivity measurements gave r_1_= 6.1 mM^−1^·s^−1^ and r_2_ = 36.9 mM^−1^·s^−1^. The high r_1_ relaxivity (1.5 times higher than Magnevist (4.0 mM^−1^·s^−1^) [138]) is attributed to the prolongation of the rotational correlation time caused by the restricted local motion. However, in vivo experiments showed that only the nanocomposites modified with a tumor-penetrating peptide (RGERPPR) allow generating a modification of the contrast in the tumor tissue. Compared to pre-injection images (100%), the T_1_ MR signal ratio was increased to 168% and the T_2_ MR signal ratio was decreased to 77.1%.

Another emerging imaging technique, magnetic particle imaging (MPI), that uses superparamagnetic nanoparticles as tracers [139,140] could also find promising development by using IO@MS NPs. However, to the best of our knowledge, only very few papers could be found dealing with IO@MS for MPI. In particular, one group showed that tracers made of silica-coated ferrite were efficient for MPI, whereas commercial PVP-coated IO NPs quickly lost their signal in PBS, rendering them unsuitable for MPI [141]. This shows that many opportunities are to be seized in the development of IO@MS for MPI applications.

### 3.2. Design of IO@MS for Magnetic Hyperthermia

#### 3.2.1. Magnetic Hyperthermia Principles and Mechanisms

Magnetic hyperthermia (MHT) refers to a therapeutic treatment that consists of heating a region of the body above the normal physiological temperature, i.e., 37 °C for the human body. Due to their more chaotic organization and the defecting architecture of the vascular supply, cancer cells are particularly sensitive to temperature elevation and thus less tolerant to high temperatures than healthy tissues. Temperatures over 41–42 °C can deeply affect their viability. Cellular functions are affected, leading to cell degradation and finally cell death or apoptosis. This method can thus be an alternative or a complement to cancer treatment. If the temperature is higher, the cell membrane ruptures and the reaction is more inflammatory, leading to tissue necrosis, which is known as thermal ablation. Hyperthermia and thermal ablation are two techniques to destroy cancerous tissues. It has been shown that moderate hyperthermia (42–45 °C) can kill cancer cells in 15–60 min and it is shortened at 4–6 min with thermal ablation (>50 °C) [142]. In this frame, magnetic nanoparticles can be used to induce heating by the application of an alternating magnetic field (AMF). Gilchrist et al. was a pioneer in the experimentation of this treatment by using magnetic particles to heat locally lymph nodes in 1957 [143]. Since then and until today, magnetic hyperthermia (MHT) therapy by the use of magnetic nanoparticles as mediators and in particular IO NPs have gained interest in the community [144,145]. In nanocomposites IO@MS, the magnetic IO core is the active part of an AMF; the silica does not interact with the magnetic field and thus no heat from it is generated. That is why the properties of IO generate heat and act as a mediator for MH are discussed. However, the silica layer can influence heat transfer.

The ability of a material to generate heat is calculated by its specific absorption rate (SAR), which is usually measured by calorimetry (but it can also be measured by magnetometric methods). It is expressed as the capacity of one gram of material to increase the temperature of the sample in a certain amount of time. $\mathrm{SAR}=\frac{\Delta T}{\Delta t}*\;c*\;\frac{m_{\mathrm{sample}}\;}{m_{\mathrm{NP}}}\;$(in W/g) where $\frac{\Delta T}{\Delta t}$ is the variation of temperature in a defined time, c is the specific heat capacity of the sample, $m_{\mathrm{sample}}$ is the mass of the sample, and $m_{\mathrm{NP}}$ is the mass of nanoparticles in the sample [145]. This value depends on the amplitude and the frequency of the applied magnetic field, as it will influence the heat generated [146]. The intrinsic loss power (ILP) is an alternative physical value, proposed to normalize the SAR value, which is defined by $\mathrm{ILP}=\frac{\mathrm{SAR}}{H^{2}f}$. This quantity allows us to compare the results obtained by the different groups and for different materials, as it is independent of the applied field amplitude and frequency. However, ILPs are not always calculated and are sparsely reported in publications. One of the main reasons is that, to be comparable, the magnetic behavior and the heat generated have to be proportional to H^2^ * f, which is not always the case. For instance, Hergt et al. [147] showed that magnetic susceptibility χ″(t) is strongly dependent on the frequency of the magnetic field. Many experimental aspects can also affect the calculated SAR, such as the geometry and the thermal insulation of the sample [145,148,149], or the measurement method [149].

**Figure 11:**
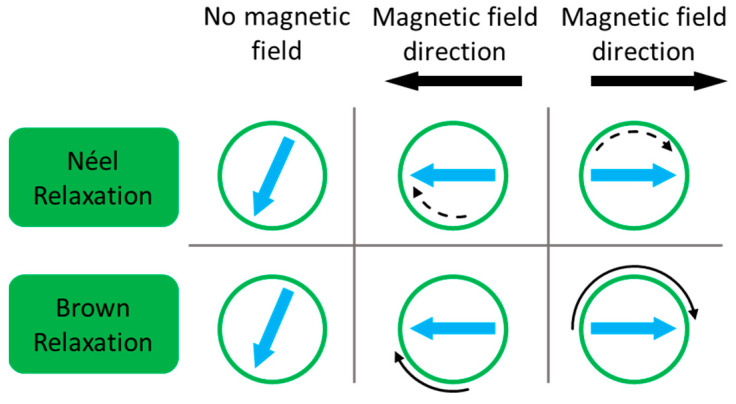
Mechanisms of heat generation in single domain superparamagnetic NPs upon exposure to an alternating magnetic field (AMF). Green circles represent NPs and blue arrows indicate the direction of the magnetic moment. In the case of single-domain MNPs, magnetic relaxation occurs in the form of internal changes in the magnetic moment direction (Néel, dashed black arrows) or physical movement (Brown, solid black arrows) as the particles attempt to align with the applied magnetic field. Adapted from Suriyanto et al. [151].

For superparamagnetic IO NPs, no hysteresis is observed on the magnetization curve and no heat loss can be generated by this phenomenon. However, as said before (Section 2.1.2 about superparamagnetism), IO NPs are set in one spin orientation and the AMF supplies the energy needed to overcome the energy barrier. Thus, the orientation of the macrospin of superparamagnetic IO NPs continuously changes at a defined frequency. Two mechanisms result in the dissipation of thermal energy upon this stimulus: the Néel relaxation, coming from the internal friction of the crystal lattice with the reorienting magnetic moment and the Brown relaxation, which originates from the viscous energy dissipation from the NP turning in the surrounding medium [150]. As a function of the composition, size, shape, crystalline anisotropy, and aggregation state of the NPs, as well as the viscosity of the surrounding medium, the relative contribution of each mechanism on the NP movement will be different. With large particles and low viscosities, Brownian relaxation dominates, whereas with small particles, highly viscous fluid or NPs (in a solid matrix, for example) trapped in Néel relaxation will be predominant. Both mechanisms can occur simultaneously, and the overall effective relaxation time of the ferrofluid will be the result of both phenomena (Figure 11).

As a result, it is important to consider the parameters influencing the relaxations mechanisms in order to design IO@MS NPs adapted for MHT and for the temperature elevation expected in the system. Several parameters play a crucial role to have effective nano-objects. These can be divided between extrinsic and intrinsic parameters.

#### 3.2.2. Main Parameters Influencing MHT Potential

##### Extrinsic Parameters

*The viscosity of the environment*. As we stated before, the contribution of each relaxation mechanism depends in part on the environment in which the NPs are settled. The viscosity of the fluid is of crucial importance because the friction of rotating NPs in this fluid participates in the heating [145]. By slowing down or blocking the NPs in more viscous media, it is possible to evaluate the contribution of Brown relaxation for heat loss. By systematically studying the influence of different parameters on the SAR values, de la Presa et al. [152] showed that by increasing the viscosity of the liquid, the heating power of large NPs decreases, whereas it remains stable for smaller NPs. This experimentally confirms that for small NPs, heat loss mainly occurs through Néel relaxation while the Brown relaxation mechanism is predominant for large NPs.

*AMF amplitude and frequency.* Dissipated heat depends on the amplitude and the frequency of the magnetic field. This means that the SAR values increase by increasing the amplitude and/or the frequency of the AMF applied. The typical range of frequencies (f) is 100–700 kHz and is between 10 and 30 kA·m^−1^ for the amplitude (H). It is commonly admitted that for clinical uses, the product H * f should be lower than 5 × 10^9^ A·m^−1^·s^−1^ [153,154] in order to avoid non-localized temperature increase due to the generation of Eddy currents [155]. This value was established in the 1980s but to date, it remains a reference and a basis for work even if the evaluation of this limit has not been the subject of recent investigations.

##### Intrinsic Parameters

The heating power of the NPs depends on their magnetic properties. MHT is influenced by the particle anisotropy, the blocking temperature, and the Brown and Néel relaxation times, but the size is also among the main parameters.

*Size.* As seen above, the size of the IO NP and especially if it is monodomain or not is of key importance. Above 100 nm, the hysteresis loss is predominant for large blocked NPs. For superparamagnetic NPs, Néel and Brown relaxation are predominant. Depending on the size, the proportion of each relaxation mechanism is different. The influence of core size has been the subject of many studies [147,156]. However, the reported optimal size differs from one publication to the other, and this mainly comes from the physical properties of the NPs and the synthesis method which can induce polydispersity and crystalline phase defects [157,158]. However, optimal values for IO cores are reported for diameters between 12 and 25 nm [58,159,160]. In a paper by Gonzalez Fernandez et al., they showed the very strong influence of the size of the IO on the magnetothermal properties. An optimal diameter of 24 nm was found to have the highest SAR. Below or above this value, the SAR decreased very rapidly [160]. This follows the expected behavior obtained from the theory described above and reported by Rosensweig in 2002 for a mechanism based on Néel relaxation in single-domain particles [146].

*Particle anisotropy***.** Magnetocrystalline anisotropy and shape anisotropy are key factors to optimize heat generation under AMF. When a magnetic field is applied, the superparamagnetic NPs tend to align in the field direction, and the energy needed to do this is called crystal anisotropy. It is primarily due to spin-orbit coupling. Among the iron oxides, magnetite has the highest anisotropy constant at 11–14 kJ·m^−3^ compared to maghemite, for which it is about 4.6 kJ·m^−3^. On the other hand, the modification of the shape is also a way to improve the MHT effect. At the surface of the NP, changes in the neighboring atoms and the crystal orientation may lead to changes in the local magnetization with respect to the surface. For instance, cubic or faceted NPs show higher SAR values than spheres. Nanocubes of 19 nm were found to have an impressive SAR value of 2453 W·g^−1^ at 29 kA·m^−1^ and 520 kHz [161].

#### 3.2.3. Core-Shell IO@MS NPs for Magnetic Hyperthermia

Core-shell IO@MS NPs are increasingly studied in the literature for magnetic hyperthermia treatment, but the influence of the silica shell is sparsely discussed [162]. Gonzalez Fernandez et al. found that the silica shell plays an important role in the heating behavior of these core-shell NPs in an AMF, by hindering the heat dissipation out of the NPs and thus decreasing the heating efficiency (lower SAR) [160]. Adam et al. showed that by increasing the pore diameter of the MS, it was possible to increase the heat outflow through the silica shell [18]. Appropriate engineering could thus attenuate the insulating nature of the silica.

Tao and Zhu [163] described the synthesis of MS shells around polydisperse IO cores ranging between 15 and 20 nm. They also reported a decrease in the heating capacity when a silica shell was added around the IO cores. Fe_3_O_4_ NPs without silica coating had a SAR measured at 16.9 W/g, whereas the IO@MS NPs had SAR values between 2 and 5 W/g when exposed to an AMF (f = 298 kHz, H = 150 G). This is due to the fact that the SAR calculated here is expressed as W per gram of NPs, which takes into account the relatively high amount of non-magnetic mesoporous silica which is not implied in the overall heating. By increasing the concentration of IO NPs embedded in the silica, SAR and heating capacities increase. Moreover, the SAR of IO and IO@MS NPs increased when the amplitude and intensity of the AMF increased. This was reported by many groups [136,164,165].

On the other hand, the MS coating layer is of crucial importance to maintain the magnetothermal properties of the IO core while adding properties of silica such as porosity, colloidal stability, and chemical versatility. An appropriate design is thus needed, and reaction conditions have to be controlled. In contrast to the previous examples, many groups reported an increase in the SAR when adding a silica layer around IO cores. This phenomenon appears mainly when the silica layer improves the colloidal stability. Hurley et al. showed that even if IO NPs had higher SAR than IO@MS in water, the behavior was the opposite in complex media mimicking body fluids. The silica layer by preventing the aggregation helps to maintain heating capacities compared to IO NPS which aggregates and sediments in these media [166]. Majeed et al. showed the same trend when comparing the SAR of IO NPs uncoated and coated with silica. Due to less clustering thanks to the silica layer, the coated NPs showed better heating performances in AMF. However, when a thicker silica layer is deposited around the IO core, SAR decreased again because of the strong thermal insulation of the core.

A silica layer around IO cores also tends to separate apart and keep these cores away one from the other. By modifying possible dipole–dipole interactions between IO NPs, this separation may have a strong influence on the heating behavior in AMF. Jiang et al. showed that monodisperse IO NPs having a mean diameter of 14 nm tend to form hexagonal close-packed structures in the absence of an external magnetic field, which is interpreted as the fact that there is no obvious dipole–dipole interaction between the NPs, and thus indicated a superparamagnetic behavior. However, they also noticed a decrease in the blocking temperature from 200 K to 100 K for non-silica-coated and silica-coated IO NPs, respectively, which still indicates that there is a decrease in the magnetic interactions between IO NPs coated with a silica layer [167]. Thanks to very good control of the thickness of the silica layer, it was possible to progressively reduce the dipolar interactions between superparamagnetic IO cores, thus making it possible to distinguish their contribution to the magnetic properties of these IO NPs. José Rivas et al. were thus able to precisely calculate the effective anisotropy constant which helps to have better predictions of the magnetothermal performances of IO and IO@MS NPs [168]. Concerning the use of ferromagnetic NPs for magnetic hyperthermia applications, dipole–dipole interactions tend to decrease the heating power of NPs in an AMF. Serantes et al. showed that these interactions modify the energy barrier and the global magnetic behavior of the NPs. They also affect magnetic susceptibility and thus hysteresis losses [169]. For this kind of system, the coating with a silica layer improves significantly the SAR, cellular uptake, and intracellular magnetic hyperthermia performances [170,171,172].

Due to their very appealing properties and biocompatible features, many IO@MS systems have been developed to perform magnetic hyperthermia therapy. Andrade et al. described the synthesis of IO@MS nanocomposites. The cores were produced by coprecipitation, and the silica layer was deposited via the Stöber method. This very easy method led to polydisperse silica-coated nanoparticles containing between one and several dozens of IO cores. Even if the structure was not well-defined, the heating capacities in water and hydrogel were good, with temperature elevations between 6 and 25 K [173]. For their part, X. Lu et al. managed to produce a very interesting microstructure made of agglomerated IO NPs with a diameter of 20 nm synthesized in a previous step. The average size of these clusters was around 260 nm, and they have hollow interior structures. Afterward, they were coated with an MS layer which was then etched in hot water in the presence of PVP. This resulted in a structure having triple porosity. The object depicted superparamagnetic behavior and a high saturation magnetization, leading to very good heating properties in AMF.

### 3.3. Design of IO@MS for Photothermal Therapy

**Figure 12:**
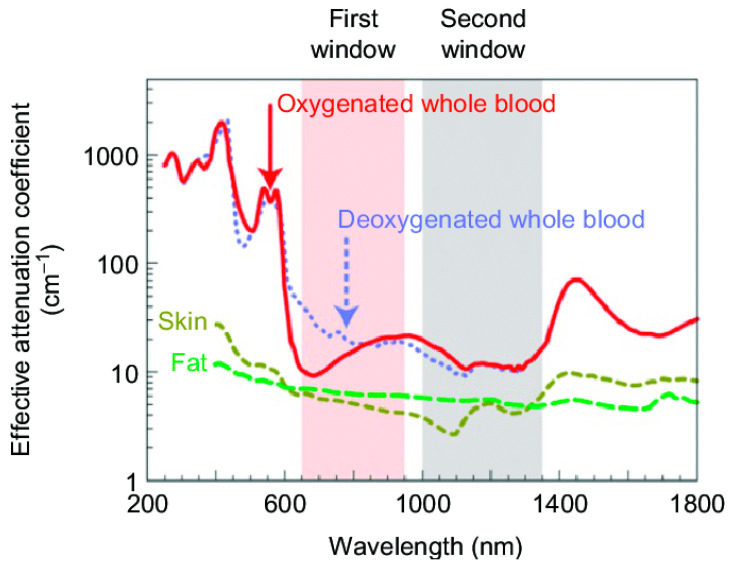
The extinction coefficient of light in different biological components of human tissue [174]. Reprinted (adapted) with permission from [174].

Photothermal therapy (PTT) is another kind of thermal treatment that is induced by the application of light and especially lasers. This method has been demonstrated to be very efficient to destroy cancerous tumors. However, the heat sink effect (heat dissipation to the environment) dissipates heat and decreases the potency of the thermal effect. Using lasers directly to ablate cancerous tumors would require high-energy setups causing damage to normal tissues. Thus, traditional laser-induced photothermal therapy has been considered to be non-reliable and limited to superficial tumors as human tissues absorb light in the visible range of the electromagnetic spectrum. Nanotechnologies are thus particularly suited to overcome these issues. To improve the efficiency of PTT, light-absorbing nanomaterials called photothermal agents have been developed. These nanoparticles convert the light into heat. The heating is thus local and surrounding healthy tissues stay at normal body temperature. The most light-absorbing tissues in the human body are the skin (melanin), the fat, water, and hemoglobin. It is thus necessary to find a range of wavelengths where these tissues are partially transparent in order to have minimal scattering and absorbance, preserve healthy cells, and penetrate the deeper possible to reach the photothermal agents. This range is called a biological window. One window is found around 800 nm and is known as the first biological near-infrared (NIR) window. A second biological window extends between 1000 nm and 1350 nm (Figure 12) [174]. In this range, NIR light can penetrate tissues up to 3 cm [175,176].

#### 3.3.1. Nanomaterials for Photothermal Therapy

Indeed, due to its simplicity and low cost, NIR light can be easily applied through a range of lasers with tunable power and wavelength. NIR laser-induced hyperthermia is developed as a minimally invasive treatment, where photothermal organic or inorganic sensitizers turn this absorbed light into localized heating at the nanoparticle scale [122,177,178,179,180,181]. Melamed et al. defined the criteria needed for a good photothermal agent: (i) minimal toxicity/maximal biocompatibility, (ii) a diameter between 30 and 200 nm to promote long circulation and enhanced tumor accumulation, (iii) the ability to absorb NIR light, and (iv) a high absorption cross-section to maximize light-to-heat conversion [182]. A vast set of nanomaterials are today available as photothermal agents of different compositions, shapes, sizes, structures, and surface coating. Among the inorganic NPs reported, gold-based nanomaterials are the most tested and represented. Indeed, metallic nanostructures and especially gold have a unique photophysical property: the local surface plasmon resonance (LSPR). When an electromagnetic wave interacts with a plasmonic material, the oscillating electric field of the radiation results in synchronized oscillations of the conduction-band electrons at the surface of the nanoparticles. Within NPs, the oscillating electrons further collide with the nuclei and as a result, energy is transferred into vibrational modes of lattice (phonon) and converted into heat [183]. At the so-called LSPR wavelength, the oscillation is maximal. This excitation state is transitory and the electrons are de-excited through a non-radiative decay, which thus generates heat. For instance, the company Nanospectra Biosciences (USA) has clinically developed photothermal ablation based on silica@gold core-shell nanoparticles for the treatment of prostate cancer, and the non-toxicity of their gold and silica NPs was demonstrated [184]. Silver NPs produce about 10 times more heat at their plasmon resonance but due to their chemical instability, gold is still favored for biomedical applications.

**Figure 13:**
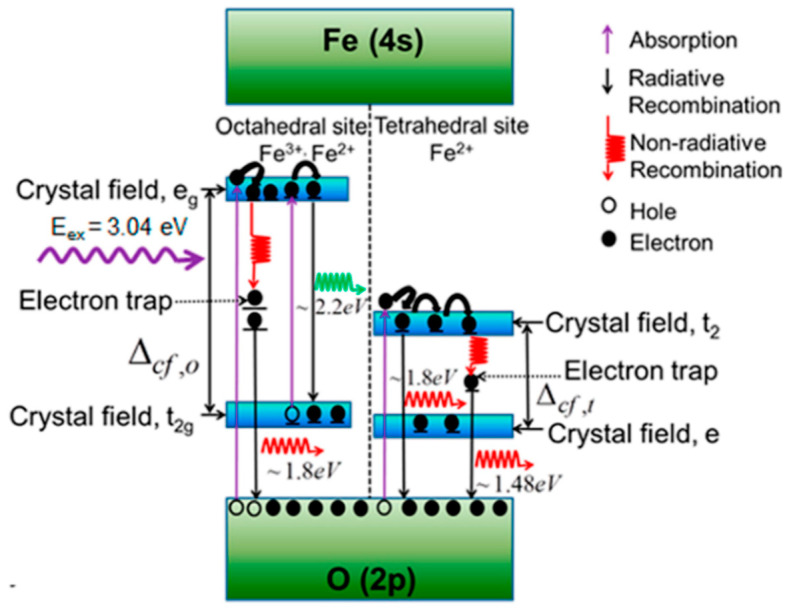
Schematic of the energy bands of the magnetite NPs system [185]. Reprinted (adapted) with permission from [185].

In addition to gold and metallic NPs, other materials have been shown to absorb NIR light. Recently, non-metallic inorganic nanoparticles and, in particular, magnetite Fe_3_O_4_, has shown to produce a photothermal effect when irradiated by NIR light [183,185]. IO NPs have been shown to be attractive NIR light mediators and to have great potential for photothermal treatment [186,187,188,189]. Espinosa et al. compared the efficiency between a PTT of gold and IO NPs. They concluded that IO NPs can compete with gold NPs at low doses with a SAR of ≈3000 and 600 W g_Fe_^−1^ at 0.05 and 5 g_Fe_ L^−1^ (1 W cm^−2^), respectively [190]. The physical mechanism which generates heat in magnetic NPs is slightly different from plasmon resonance. For these electronic transition materials, photothermal transduction is led by transitions of the electrons from the valence band to the conduction band. When the electron relaxes back, heat or light is generated. Figure 13 details the approximate band structures of Fe_3_O_4_ NPs between the valence band of the O(2p) to the empty Fe(4s). Radiative and non-radiative decays are both involved. The exact mechanism is still unclear and it certainly results from several processes, such as the fast decay of electrons and the release of phonons instead of photons [185].

#### 3.3.2. Parameters Influencing Photothermal Effect

We defined the criteria for an ideal photothermal agent. It is important that NPs interact optimally with NIR light to convert it into heat and to exploit the biological tissue’s windows. They also have to be non-toxic and stable enough to reach the target area (tumors). Gold NPs are very efficient to convert NIR light into heat, and Zhang et al. [191] showed how the shape of the particles is a fundamental parameter to optimize the photothermal effect. Anisotropic NPs generate a more defined charge separation when their SPR is excited, a plasmon resonance wavelength shifted towards NIR regions and by optimizing the intensity of SPR, the heat released can consequently be more efficient.

Furthermore, size is always a fundamental parameter when dealing with NPs, as surface properties predominate over volume effects. With careful control of the synthesis, it is possible to fit the absorption wavelengths with the incident wavelength emitted by the laser or diode; thus, the heat production is also maximized. This is true for plasmonic NPs. However as detailed above, the mechanism of photothermal conversion of IO NPs is very different, as it primarily originates from the transitions of electrons of neighboring Fe ions within the lattice. As a consequence, as long as the emitted wavelength is in the NIR range, the mid-bandgap states trap the excited “hot” electrons and produce phonons that carry heat [192].

When optimized, PTT is a very local therapy, and the location of the NPs has an impact on the treatment efficiency. In vitro experiments have compared the efficiency of PTT on extracellular and intracellular treatments. They showed that PTT was more efficient when gold nanorods were directly internalized by the cells than when gold nanorods were outside of the cells [193]. PTT results in different physiological and biological modifications in the tumor tissue which can improve therapeutic effects and enhance the efficacy of secondary treatments, such as chemotherapy. The localized heat enhances the permeability of cell membranes and tumor vasculature, thus allowing a better drug uptake [194,195].

Photothermal therapies use continuous or pulsed wave lasers. These two types are very different and induce different mechanisms of damaging cells as they have different time profiles and intensities. Optimum laser intensity depends on the cell type, the used photothermal agent, and the cell environment. Efficient treatments are carried out with a power laser range from 0.5 to approximately 100 W·cm^−2^ [183].

#### 3.3.3. Photothermal Therapy with IO@MS NPs

It has been stated above in the previous section that the coating of IO with a silica shell could significantly improve the efficiency of MHT as well as bring additional properties, such as colloidal stability and surface chemistry versatility. Quite logically, several teams also tested IO@MS NPs as PTT agents. Adam et al. coated monodisperse IO NPs with a stellate mesoporous silica shell. The photothermal properties of aqueous dispersions when exposed to an NIR light laser (1064 nm) were quantified as a function of laser power and NP concentration. They showed a strong decrease in the photothermal SAR values when the concentration increased; from 2014 to 453 W g^−1^ for concentrations of 0.013 and 0.26 mg [Fe_3_O_4_] mL^−1^, respectively, at a fixed laser power of 1 W cm^−2^. This behavior was explained by a limitation of the illumination penetration depth when the concentration of NPs increases due to light absorption. As the incident light comes from one direction and the sample has a certain thickness, there is a gradient of incident power inside the sample and on average, the NPs can absorb it. Moreover, they investigated the influence of the laser power and showed an exponent-like growing evolution. This effect was also explained by a better penetration of the laser beam in the dispersion [196]. The concentration dependency of photothermal SAR was also shown by Nemec et al. [197]. In this study, they investigated the effects of silica encapsulation of IO NPs and clustering on both magnetic hyperthermia and photothermia. Indeed, it is well-known that endosomal internalization of IO NPs, which leads to clustering, has a negative effect on MH [190]. Here, they showed that clustering does not affect photothermia efficiency and that silica coating even improved heating capacities [197]. As the research around the use of Fe_3_O_4_ NPs for photothermal therapy is quickly growing and spreading in different laboratories around the world, it is necessary to define a standard way to evaluate the SAR because, as stated above, many parameters can interact with the results. In 2023, de la Presa et al. [198] proposed to utilize the commercial IRA 980B as a reference probe to report the heating efficiency of iron oxide colloids under infrared irradiation. They also made recommendations about the important parameters to report and the way to conduct the measurements.

By assembling silica-coated clusters of SPION into nanochains, Kolosnjaj-Tabi et al. synthesized an original structure having a strong magnetic responsiveness and a very efficient photothermal agent. Upon NIR irradiation, these nanochains have not only efficient cytotoxic photothermal properties due to the heat generation, but they were also able to locally melt the collagen matrix. This double action on cancer cells and their environment could be very promising in the development of cancer therapy [199]. Other original structures combining IO and silica for PTT were reported. Ji et al. developed NPs of IO@MS coated with a gold nanoshell. They showed that this nanoobject is efficient both as an MRI T_2_ contrast agent and as a photothermal agent. Thanks to their magnetic properties, the NPs can also be directed by a magnet to a disease site, for example [200]. Huang et al. described the synthesis of yolk-shell silica-coated hollow carbon nanosphere-encapsulating Fe_3_O_4_ NPs. These NPs demonstrated an efficient photothermal conversion at 808 nm. After intravenous injection, a magnetic field was applied in order to concentrate the NPs at the tumor site, followed by 10 min of exposure to an NIR laser. This formulation showed a very good ability to kill cancer cells and treat subcutaneous tumors [201].

Photothermal therapy is not only developed for anticancer applications. Other health issues can be addressed using this treatment, such as microbial resistance. Nanoplatforms for antimicrobial applications have been developed in recent years. They are mainly based on gold [202], metal sulfides, and polymer or carbon nanomaterials [203,204]. Regarding iron oxide silica core-shell NPs, no system is reported. However, several publications deal with the use of iron oxide NPs coated with a polymer layer [205,206,207], with a gold shell [208], or coated with alumina [209]. For its part, silica has been reported for such applications as a shell around gold NPs [210,211]. Thus, there are opportunities to explore this field for IO@MS NPs.

### 3.4. Design of IO@MS as a Carrier for Drug Delivery

As detailed above, silica coating has several advantages. (i) They provide not only stability to the IO cores in an aqueous solution but they also avoid interparticle interactions leading to aggregation. (ii) Silica shell structural properties (thickness, porosity) can be easily controlled. (iii) IO@MS possesses good biocompatibility. Silica coating is also a great chemical platform to bind—covalently or not—therapeutic molecules and to load them into and onto the pores, and a plethora of strategies have been developed. A lot of parameters influence drug loading. The most important are NP functionalization and surface charge, the nature of soaking media, the pH of the soaking media when they are an aqueous solution, and the presence of gatekeeper molecules or coupling agents for covalent bonding.

To describe the ability to load a drug, three main parameters are generally used in the literature: feed weight ratio (Fwr), drug loading content (DLC), and drug loading efficiency (DLE).

$$\mathrm{Fwr}=\frac{\mathrm{mass}\;\mathrm{of}\;\mathrm{drug}\;\mathrm{given}\;\mathrm{in}\;\mathrm{soaking}\;\mathrm{media}}{\mathrm{mass}\;\mathrm{of}\;\mathrm{carrier}}*100$$

$$\mathrm{DLC}=\frac{\mathrm{mass}\;\mathrm{of}\;\mathrm{drug}\;\mathrm{loaded}}{\mathrm{mass}\;\mathrm{of}\;\mathrm{carrier}}*100$$

$$\mathrm{DLE}=\frac{\mathrm{mass}\;\mathrm{of}\;\mathrm{drug}\;\mathrm{loaded}}{\mathrm{mass}\;\mathrm{of}\;\mathrm{drug}\;\mathrm{given}\;\mathrm{in}\;\mathrm{soaking}\;\mathrm{media}}*100$$

First of all, Cotica and coworkers [212] compared the cell viability in presence of IO and IO@MS NPs (aimed for DOX loading) and their interactions with cells. They established the importance of a silica coating to improve the biocompatibility of these nanoobjects. 

The loading of DOX in slightly basic media can considerably increase the DLC. Dmitrienko et al. [213] used a sodium borate buffer at pH 8.0 to load DOX on silica nanoparticles and they obtained a DLC of up to 25.8%. By grafting Nylon-6 chains on the surface, the DLC increased to 49.3%. In another publication, Demin and coworkers [214] also loaded DOX on core-shell IO@MS NPs. These nanocomposites were coated with PEG molecules and they obtained a DLC of around 15%. They showed that, in acidic media, the release was significantly increased. Shao et al. [215] managed to load about 20 wt% of DOX in the bare NP pores and observed that at acidic pH (pH = 5.5) a lot more DOX is released compared to pH = 7.4. This pH-dependent behavior can be explained by the fact that the main driving force for DOX loading should be electrostatic interactions. However, for most of these systems, DOX is bounded only through these weak interactions with the NPs, and a lot of leaching at room temperature and physiological pH is observed, which is undesirable for biomedical applications.

The formation of covalent bonds such as urea, ester, or amide bonds to covalently conjugate the DOX on the NPs considerably improve DLCs and decrease drug leaking compared to only hydrophobic, van der Waals, or electrostatic interactions. For example, Li et al. [216] showed that the DLC increased from 13% to 39% by connecting DOX to the NPs through a urea bond by the reaction of isocyanate-silane with the amine group of a DOX. Recently, Waters and coworkers [217] compared three different techniques to load a model drug onto ferrite NPs coated with a silica shell. The electrostatic loading leads to constant leaching, which is not convenient. The second technique used the grafting of the drug model through an ester covalent bond which is known to be acid labile. However, the rate of payload release was very slow, at neutral and acidic pH, and steady leaching from the nanocarrier even at neutral pH was present. Finally, they linked the drug model to the carrier through an amide bond. They observed a very limited free leaking (3.6% after 24 h) but in the presence of a low frequency AMF (27–35 Gauss, 100 Hz), up to 80% of the loaded payload was released after 30 min exposure to the AC magnetic field and ≈100% was released after 8 h post-AC-field exposure.

Different strategies of drug gatekeeping and triggered release at the surface of IO@MS NPs were described in the literature. Peralta et al. [218] grafted a PNIPAM-co-MPS polymer on IO@MS NPs. During the polymerization, the comonomer MPS, 3-(Trimethoxysilyl)propyl methacrylate) acts as an anchor group on the silica surface. The empty pores of the silica shell were filled with the ibuprofen drug model. At 40 °C, the lower critical solution temperature (LCST) of the polymer, about 5 times more drugs were released as compared to 20 °C, thus showing the gatekeeper role of the polymer for thermoresponsive release.

Moreover, the porosity allowed good drug loading, and the application of AMF was shown to trigger drug release [219]. Saint-Cricq et al. [220] modified IO@MS with APTES, and then azo-PEG was coupled to the surface. Under AMF, the azo bonds break and the drug (here, rhodamine 6G as a proof-of-concept dye) was released. Zhu et al. [221] reported the use of double-strand DNA as a gatekeeper to cap IO@MS NPs. Upon heating, the denaturation of the dsDNA unleashed the DOX stored in the pores. Guisasola et al. produced IO@MS NPs grafted with an engineered thermoresponsive polymer as a gatekeeper, which is able to release a preloaded drug when the temperature reaches 43 °C. They demonstrated the “hot spot” effect which means that, upon AMF, the central IO core heats up and the heat diffuses to the outside of the core-shell NPs. Thanks to this effect, they showed that it was possible to trigger the release of a drug without global heating; local heating at the scale of the NP is enough [222,223]. Patil-Sen et al. showed the possibility to use lipids to coat IO@MS to have a very good biocompatible formulation. This formulation was also able to release doxorubicin (DOX) when exposed to AMF [224]. Indeed, IO@MS NPs are optimal to combine magnetic hyperthermia and chemotherapy, and much research is made in that direction [225]. A lot of papers show a very good synergetic effect of MHT and drug delivery on the killing efficiency of cancerous cells [142,222,226]. A lot of papers suggest various formulations loaded with drugs. In 2015, Zhu and Tao [221] and Xu et al. [227] used both carboxylated DNA grafted on the surface of amine-modified IO@MS as a thermoresponsive gatekeeper. Above 37 °C, the DNA cap swells, and the preloaded drug is released. This temperature elevation could be obtained by using an AMF (f = 409 kHz, H = 90–180 G).

More recently, Horny et al. [228] presented the use of IO@MS core-shell NPs for microRNA detection. The NPs were used to graft DNA probes hybridized with their complementary DNA targets. A magnetic field signal (535 kHz and 10.56 kA m^−1^) was applied for a few minutes to measure the SAR, which was found to be between 64 and 82 W per gram of magnetic material, depending on the size of the nanoobject. In order to dissociate the DNA strands, they showed that local heating induced by AMF was almost as efficient as global heating at 95 °C while keeping the solution at 28 °C, which was crucial for biological media. A wide variety of organic coatings of silica layers were described in the literature to bring enhanced biocompatibility, drug cargo, and gatekeeper properties [224,229,230].

Disulfide bonds are also interesting gatekeepers, as they are stable in blood circulation and their degradation is only triggered by reduced glutathione or thiols and, in particular, by glutathione reductase (GSH), whose concentration is usually twice as high in tumors compared to normal tissues [231]. The use of IO@MS NPs also permits the development of nanoplatform coupling drug delivery with imaging properties and/or additional modes of therapy. Among the various existing potential coatings, the stabilization of iron oxide or IO core-shell NPs with proteins is of high interest to limit toxicity and prevent immune system response [232,233,234]. For instance, coatings with enzymes may allow ensuring biocatalytic activities while the use of human serum albumin is relevant to limit opsonization and to increase blood circulation time. For example, Ménard et al. used human serum albumin (HSA) as a protein gatekeeper around doxorubicin-loaded IO@MS. The drug was released after contact with cancer cell spheroids through a hypothesized enzymatic degradation of the protein layer. Thanks to the central IO core, these platforms also showed excellent contrast agent properties for T_2_-MRI [17]. Adam et al. also studied the combination of drug delivery with photothermal therapy thanks to the good photothermal properties of IO NPs when exposed to near-infrared (NIR) light [196].

### 3.5. Nanothermometry

When speaking about MHT or PTT, it is the temperature of the whole macroscopic sample, in a tube, in vitro cell culture, or in vivo tissue that is generally considered. However, the heat dissipates locally from the NPs to their environment. Knowing the temperature into or at least at the surface of these nano-objects can be of crucial importance when the NPs carry thermally sensitive molecules such as some biomolecules, proteins, peptides, DNA, and RNA. When the NPs are in direct contact with cells, the local temperature can also have a huge impact on the surrounding cellular structures, such as the cell walls or the nearby organelles, even if the macroscopic temperature does not generate overheating. This local heating or “hot-spot effect” can thus be advantageously used for biomedical applications [230]. Indeed, the temperature profile of NPs can be used to induce a thermal trigger for drug delivery without damaging the area of interest; for example, in the case of the activation of biological functions [235] or the delivery of sensitive siRNA to specific cells that do not have to be thermally killed. Potential side effects are limited. However, evidence of the local heating involved in the nanoscale hot spots is not an easy task, as it cannot be measured with usual macroscopic tools, such as thermometers or infrared cameras.

Several molecular or nano-tools have been developed to obtain proof of the local temperature state. Thermosensitive chemical reactions are the most developed way to sense the local temperature. Zink and coworkers reported many systems and ways to probe them, such as the retro Diels-Alder reaction, which was used and monitored by MHT on superparamagnetic doped iron oxide@mesoporous silica core-shell NPs [236]. Pores filled with fluorescein were closed by bulky cyclodextrins which were bound to adamantane groups by supramolecular groups. The temperature was set at 0 °C. To obtain an equivalent release of fluorescein without a local trigger, the reaction should be performed at 65 °C, thus demonstrating the thermal local effect of AMF stimulation. Thermoresponsive block copolymers were also used as nanothermometers on magnetic NPs. PNIPAM was copolymerized with a fluorescent monomer and when the lower critical solution temperature (LCST) was reached, the polymer shrunk, and a change of fluorescence was observed [237]. Griffete et al. [238] showed that polymerization can be induced from the surface of magnetic NPs by the application of AMF. This polymerization should occur at temperatures above 60 °C but with AMF, they obtained it under a macroscopic temperature of 31 °C, demonstrating the local effect. In another example, IO@MS NPs loaded with fluorescein and coated with a thermoresponsive polymer shell were stimulated by AMF. Even if the polymer was at a distance of 20 nm away from the nanocarrier, LCST transition was observed under AMF [223]. These results demonstrated how the local thermal effect can become of great interest for the design of the next generation of magnetic NPs for nanomedicine applications.

**Figure 14:**
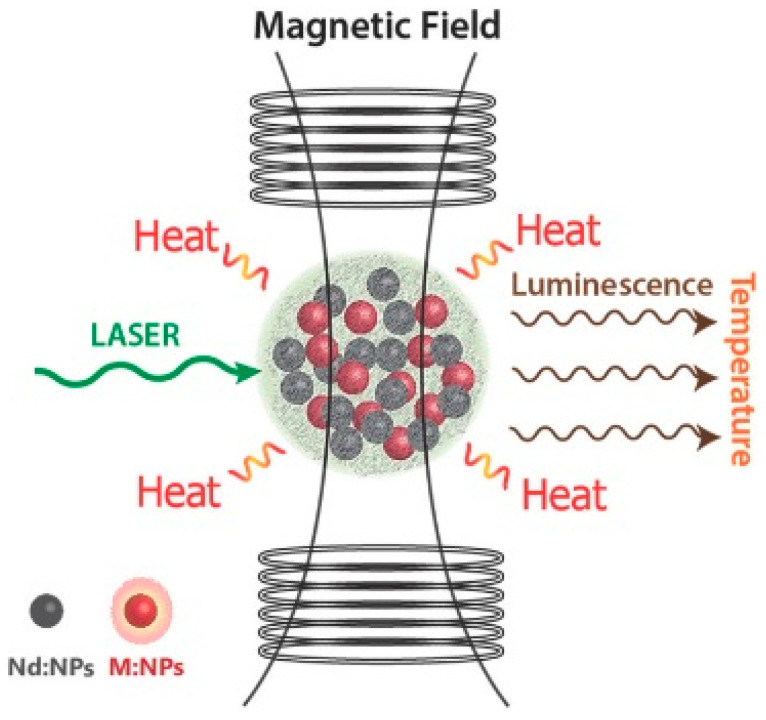
Scheme to illustrate an experiment of nanothermometry under a magnetic field using a luminescent nanothermometer [239]. Reprinted (adapted) with permission from [239].

Thus, the “hot-spot” effect was first explored by macroscopic observations resulting from the nanoscale-derived effect. Fewer attempts have been made to directly sense the temperature profile into and outside the nanoparticle. Jaque et al. [239,240] reviewed the use of optical techniques and, in particular, the use of luminescent nanothermometers, such as quantum dots, whose luminescence is dependent on the temperature (Figure 14). Dong and Zink [241] used up-conversion nanocrystals NaYF_4_:Yb^3+^,Er^3+^ whose fluorescence emission spectrum is modified when the temperature is changed. These latter nanocrystals were incorporated inside a mesoporous silica nanoparticle together with superparamagnetic IO NPs. Inside the silica matrix, a more rapid temperature increase of 45 °C was detected as compared to a bulk temperature increase of 20 °C. This concept is of prime importance to elucidate temperature profile distribution near the hot spot. Pellegrino et al. [242] demonstrated that it is possible to measure a temperature gradient as a function of the distance of the NP hot-spot surface. For this, they used a fluorescent dye linked by an azo bond and spaced by various length chain PEG linkers to the IO NP. They showed that the temperature increase depends on the amplitude of the magnetic field (magnetic power). More importantly, their results indicated that the local temperature is 50 °C higher than the macroscopic temperature at the surface of the NPs, while the difference between these two temperatures falls a few nanometers (2–3 nm) to zero.

Optically activated NPs (for PPT application) could use exactly the same kind of nanothermometers and temperature-sensing methods. The measurement of temperature profiles on the nanometer scale is today still very challenging, as specific setups are sparsely available and fluorescence shift can be very sensitive to numerous environmental changes. However, the impact on biological applications could be very promising, in particular for the control and monitoring of magnetic and/or light-induced hyperthermia.

## 4. Biological Applications of IO@MS Core-Shell NPs

### 4.1. IO@MS NPs—In Vitro/In Vivo Cancer Therapy Applications

#### 4.1.1. Interactions of NPs with Living Systems

Nanotechnology is quite a recent research field, and it has changed the way therapeutics are designed and formulated. Conventional injectable therapeutics exhibit some major disadvantages such as lack of selectivity, low aqueous solubility, low bioavailability, and a rapid fall in the plasma concentration due to rapid clearance of the drugs. Injectable treatments, which can deliver a sustained controlled release, would allow reducing repeated administrations and hospitalizations by maintaining therapeutic drug levels in the plasma. The size of nanoparticles and their high area-to-surface ratio, targeting ability by surface functionalization, receptor attachment, or EPR effect make these objects very suited to treat diseases such as cancers, autoimmune diseases, or diabetes. Enhanced radiotherapy, hyperthermia, targeted drugs and DNA/siRNA delivery, development of contrast agents for MRI, and CT imaging are among the new possibilities offered by injectable nanoformulations. The advantages of such injectable nanoformulations over conventional therapeutics are numerous but can also have some limitations such as difficult in-depth penetration in tumor tissues, possible early opsonization and phagocytosis, and difficult cell internalization. These latter effects will depend on the particle size, shape, surface charge, and stabilizing ligands [243].

Indeed, to bring magnetic nanoparticles further into concrete biomedical and therapeutic applications, it is necessary to have biocompatible formulations. In this frame, IO and silica are two materials of choice among inorganic materials. Arami et al. reviewed the studies dealing with in vivo toxicity of IO NPs. Many factors can influence the toxicity of the NPs, such as the mode of administration and variations between animal models or humans, but also the characteristic of the NP itself, such as the surface charge, the size, the morphology, the type of coating, and so on. However, three major characteristics of IO NPs should guarantee their clinical success: pharmacokinetics, short- and long-term tolerability, and theranostic functionality in the desired organ [162,244]. Coated IO NPs are usually less toxic than naked particles and the coating determines the biocompatibility [244]. Silica is “generally consider as safe” (GRAS); amorphous silica is even an FDA-approved food additive. Down to the nanoscale, some toxicity could appear due to the interaction of cells with the NPs. However, toxicology studies of silica-coated NPs have been very limited. Most of the MTT assays show that IO@MS are non-toxic due to the biologically inert surface, and silica provides a stable protective layer against oxidation and reactive species [245].

Further, R. Wang et al. recently investigated cell uptake by coupling different methods. They demonstrated that internalization is a time- and concentration-dependent phenomenon. The NPs were efficiently internalized into human osteosarcoma MG-63 cells. This complete study showed that the uptake appeared between 0.5 and 2 h and that most of the NPs were located in lysosomes [246]. This information is very important because a lot of formulations in the literature displayed a pH-dependent drug delivery. Thus, it could help to improve the design of IO@MS for DD applications.

#### 4.1.2. Various Applications of IO@MS Core-Shell NPs for Cancer Therapy

In particular, core-shell NPs IO@MS, thanks to their high loading capacity, versatile surface chemistry, and intrinsic properties can find a variety of biomedical applications, and numerous publications report their in vitro and in vivo promising applications for cancer therapy [243,247,248,249]. Table 1 summarizes different following examples. 

*Dual drug delivery.* Recently, Sanchez-Salcedo et al. [250] investigated the simultaneous delivery of two different molecules, daunorubicin and anti-TWIST siRNA, from IO@MS core-shell NPs coated with polyethyleneimine (PEI) as anchoring layers for deposition of zwiterrionic groups. This construction showed excellent low-fouling protein adsorption and, under AMF stimulation, the co-release of the drugs resulted in improved synergistic cytotoxicity of Ovcar8 (ovarian cancer cells).

*Drug delivery combined with MHT.* Pon-on et al. [251] developed magnetic silica nanoparticles encapsulated in a dual pH and a temperature-responsive chitosan biopolymer NP (chitosan-g-NIPAM). Thanks to the superparamagnetic IO core, AMF stimulation can trigger the release of a DOX. A burst release is obtained at pH = 4 at 45 °C, whereas at physiological pH and temperature, the release was low. The cytotoxicity of the DOX is decreased when it is encapsulated inside the nanocomposite compared to the free DOX. Gao et al. [137] described the synthesis of IO@MS modified with a tumor-penetrating peptide and loaded with DOX. In vitro results showed the significant role of the conjugated peptide by enhancing cellular uptake and cytotoxicity of the NPs. In vivo experiments also showed a better accumulation in tumor tissue which led to an improved MRI signal and antitumor effect of DOX-loaded NPs.

*Targeting ligand*. The local concentration of magnetic material is crucial to have optimal localized heating when AMF is applied. Thus, the use of targeting ligands is also a very promising approach to concentrating the NPs at the disease site. Lin et al. grafted folic acid at the surface of IO@MS to enhance tumor internalization [252]. Legge et al. conjugated the surface of IO@MS NPs with antibodies to target integrin αvβ6, a well-characterized oral squamous cell carcinoma biomarker. They showed that they were able to target αvβ6 overexpressing cells and thermal therapy through AMF application, significantly increased the killing of the targeted tumor cells compared to the control cells [253]. The efficiency of IO@MS to kill tumor cells lies also in the good internalization of the NPs, as shown already in 2010 by Saavedra et al. [254] Further, Avedian et al. [255] synthesized IO@MS NPs coated with folic acid-modified PEI and used for delivery of Erlotinib. They observed that PEI acts as a pH-sensitive coating and the presence of folic acid increased the cytotoxicity for HeLa cells.

*Blood–brain barrier crossing*. Glioma is the most lethal type of cancer which accounts for the majority of deaths and with very poor survival rates. Glioma treatments are mainly limited by the fact that they involve the crossing of the blood–barrier barrier (BBB), which is poorly permeable to the drugs [256]. Hegganvar et al. [257] developed an in vitro BBB model of human primary glioblastoma cells (U87 MG). They synthesized BBB-permeable nanoparticles consisting of IO@MS loaded with DOX and conjugated with a modified Pluronic F-127 bearing at its end tip transferrin (Tf) to have a sustained and targeted release of anticancer DOX. The cytotoxicity assay of this nanocomposite clearly showed a lower IC_50_ than non-loaded NPs against U87 MG cells, and thus efficient anticancer activity. Under a magnetic trigger, the nanocomposite enhanced its permeability across human brain microvascular endothelial cells, which facilitates DOX uptake.

*Gene therapy*. Gene therapy by DNA/siRNA delivery has a huge potential in cancer therapy, as it has unique functions, such as the knockdown of targeted genes or specific triggering of other genes. Xiong and coworkers [258] developed in this context an IO@MS core-shell NP with large pores (12 nm) in order to load siRNA and release it under AMF. The silica shell was modified by aminosilane and the global nanocomposite was coated with acid-labile tannic acid to serve as pH-responsive coating. The study showed a high loading capacity for siRNA (up to 2 wt%) and an enhanced release when a magnetic field is applied. Tannic acid provided stability and siRNA was successfully delivered into the cytoplasm of KHOS (human osteosarcoma) cancer cells in vitro in a pH-responsive manner.

*Immunotherapy.* Recently, immunotherapy has been expanding rapidly in the biomedical community. It is based on the strengthening or the suppression of the patient’s immune system to fight disease and, in particular, to treat cancers. Zheng et al. [259] developed a nanoplatform for immunotherapy by using IO@MS coated with PEG and filled with cytosine-guanine containing oligodeoxynucleotides (CpG ODN), which can be recognized as danger signals by the immune system. However, to date, it is difficult to use free CpG ODN due to unfavorable in vivo biodistribution, a lack of specificity, and poor cellular uptake. The APTES functionalization and PEGylation allow high CpG loading capacity. They managed to activate macrophages and inhibit tumor cells when combined with chemotherapeutics while exhibiting negligible cytotoxicity in vitro. In vivo, these nanocomposites showed excellent immuno-stimulating activity.

**Table 1:** Table summarizing IO@MS NPs used as different multimodal platforms.

Application	Nanocomposite	Functionalization	Active Molecule	Reference
Dual drug delivery	Fe_3_O_4_@MS	Polyethylenimine + 2-methacryloyloxyethyl phosphorylcholine	siRNA and daunorubicin	[250]
Drug delivery combined with MHT	Fe_3_O_4_@MS	Chitosan-g-*N*-isopropylacrylamide	DOX	[251]
Drug delivery, dual MRI +cell targeting	Fe_3_O_4_@SiO_2_@mSiO_2_	Gd-DTPA	peptide RGERPPR and DOX	[137]
MHT + radiosensitizer +cell targeting	multicore Fe_3_O_4_@SiO_2_	/	L-selenocystine + Folic acid	[252]
MHT + antibody- targeting	multicore Fe_3_O_4_@SiO_2_	glutaraldehyde	Anti-αvβ6 mouse monoclonal antibody	[253]
Targeted drug delivery	Fe_3_O_4_@MS	polyethyleneimine	Folic acid and erlotinib	[255]
BBB crossing + drug delivery	Fe_3_O_4_@MS	APTES + Pluronic F-127	DOX and transferrin	[257]
Gene therapy under AMF	Fe_3_O_4_ nanoclusters@large pore MS	APTES + Tannic acid	siRNA	[258]
Immunotherapy	Fe_3_O_4_@MS	APTES+PEG	CpG ODN	[259]

### 4.2. Smart Scaffolds Using AMF and/or NIR Light as Trigger

One of the main advantages to use remote external stimuli (magnetic fields, electric fields, or light) is the possibility to activate on demand the nanocomposite, in particular, to trigger the release of drugs or heating in a precise zone of the body. It also allows for sustained drug delivery and thus maintains therapeutic activity over a long time compared to the burst level of drugs by intravenous or oral administration [260]. To overcome the problem of circulating NPs as explained above, some examples of smart polymer scaffolds (hydrogels, electrospun fibers) responding to AMF and/or NIR light are detailed here, in particular for drug delivery and cancer treatment [261].

As detailed above, Fe_3_O_4_ magnetite NPs placed in an appropriate AMF generate heat in their surrounding environment. Satarkar et al. [262] demonstrated the possibility to use this magnetothermal effect for remote drug delivery applications. They loaded superparamagnetic IO NPs in a thermosensitive PNIPAAm-based crosslinked hydrogel with vitamin B12 and methylene blue. They were able to trigger the release of the drugs. By applying AMF for a few minutes, the temperature raised above the LCST, resulting in gel shrinkage. Campbell et al. [263] prepared a subcutaneous injectable hydrogel whose crosslinking takes place by the condensation of aldehyde-functionalized dextran with IO NPs functionalized with hydrazide-modified PNIPAAm. Gelation is rapid when both components were mixed. The gel was biocompatible in vitro and in vivo, and remote and controlled drug release was shown when AMF was applied. On the other hand, Kim et al. [264] synthesized PNIPAAm electrospun nanofibers loaded with DOX and IO NPs. After AMF application, the fibers deswelled and released DOX. It successfully induced the death of human melanoma cancer cells (COLO 679) by the synergetic effect of hyperthermia and chemotherapy. More advanced biological studies were conducted by Xie et al. [265], who injected into mice a crosslinked chitosan-PEG hydrogel loading with DOX, docetaxel, and IO NPs. This gel showed self-healing and thermoresponsive behavior while being biocompatible. After AMF, the heating combined to the release of both drugs, which resulted in very efficient synergetic antitumor action in vitro and in vivo.

## 5. Conclusions

Core-shell nanocomposites, which exhibit very appealing properties, offer promising applications as smart nanoplatforms for cancer therapy. Moreover, by combining the features of at least two materials, they extend the possibilities to perform dual therapy for cancer, which is a determining step to go further in the treatment of this disease. In this state of the art, we showed that IO@MS are very well-suited biocompatible nanoplatforms to perform hyperthermia treatment induced either by AMF or NIR, combined with drug delivery and MRI.

Nevertheless, there is always progress to be made in the design of these nanoplatforms to optimize their antitumoral action and to make them safer. Before further translation into clinical trials, their physicochemical behaviors under external fields can still be improved and better characterized. In particular, the control of the heat dissipation from the core of the NP to the external environment is fundamental for efficient hyperthermia or thermal ablation applications and to understand the therapeutic mode of action of the nano-objects when they are in tissues and cells. Furthermore, the amount of drug delivered to the target sites remain, in most cases, quite low, and that is probably because the interactions of the drug with the pore walls of the silica carrier are not completely controlled. Finally, injection and dissemination of NPs in the human body stills remain a sensitive issue that strongly hampers their application in clinical uses, and this should be considered for further development of nanotechnologies in the medical field.

In our opinion, there are probably two main challenges to overcome to foster the applications of iron oxide @ silica core-shell nanomaterials:

(1) The first topic that should be explored in the future with such core-shell is to investigate their biodegradability in different biological mimicking fluids, cells, and their biological fate in vivo. Iron oxides are reported to be rapidly degradable once internalized by cells, but the degradation fate of the silica shell requires specific investigations according to its intrinsic features: thickness, morphology/size of the pore, Si-O-Si crosslinking, aggregation state, and surface functionalization but also other extrinsic parameters, such as temperature, local pH, flow dynamics, and NP concentration in the buffer or the tissue.
(2) Another topic of interest is the control of the photothermal or the magnetothermal dose delivered by the IO@MS core-shell NPs as a function of the core material and of the silica shell features. Indeed, the silica shell may have a critical role either as a thermally insulating or conductive layer to adjust the effects of treatments and to avoid thermal denaturation of potentially fragile loaded therapeutics, such as siRNA or therapeutic proteins. Designing engineered silica shells with various shell features for local thermal dose control and understanding the influence of this silica shell on the thermal transfer through physical modeling studies are important investigations to conduct. This would allow us to improve multimodal treatments that may be achieved by these nanoplatforms.

There would be also other complementary perspectives to envision, such as (i) studying the influence of the surface functionalization and other physicochemical parameters (pH, ionic strength, the nature of the biological buffer) on the drug loading and the drug release behaviors and (ii) investigating routes for the design of original supramolecular hydrogels, in which the NPs would be embedded in order to create smart implants able to deliver drugs under external triggers.
